# A Comprehensive Review of Bovine Colostrum Components and Selected Aspects Regarding Their Impact on Neonatal Calf Physiology

**DOI:** 10.3390/ani14071130

**Published:** 2024-04-08

**Authors:** Flávio G. Silva, Severiano R. Silva, Alfredo M. F. Pereira, Joaquim Lima Cerqueira, Cristina Conceição

**Affiliations:** 1 Veterinary and Animal Research Centre (CECAV), Associate Laboratory of Animal and Veterinary Science (AL4AnimalS), University of Trás-os-Montes e Alto Douro, Quinta de Prados, 5000-801 Vila Real, Portugalcerqueira@esa.ipvc.pt (J.L.C.); 2 MED—Mediterranean Institute for Agriculture, Environment and Development & CHANGE—Global Change and Sustainability Institute, Department of Zootechnics, School of Science and Technology, University of Évora, Pólo da Mitra Apartado 94, 7006-554 Évora, Portugal; apereira@uevora.pt (A.M.F.P.); cristinaconceicao@uevora.pt (C.C.); 3 Center for Research and Development in Agrifood Systems and Sustainability, Polytechnic Institute of Viana do Castelo, Agrarian School of Ponte de Lima, Rua D. Mendo Afonso, 147 Refóios do Lima, 4990-706 Ponte de Lima, Portugal

**Keywords:** bioactive components, cell, gut maturation, nutrition, microbiome, passive immune transfer, thermoregulation

## Abstract

**Simple Summary:**

Colostrum is the “starter kit” for every calf, containing nutrients, bioactive components, cells, and microorganisms that provide energy and protection against pathogens and contribute to the maturation and development of the gastrointestinal tract. Components such as fatty acids, whey proteins, oligosaccharides, immune cells, and microorganisms are present in colostrum and are essential for the health and well-being of the newborn calf, so its importance should not be underestimated. In this review, the variability of these components, as well as their impact on selected aspects of the newborn calf’s physiology, are discussed.

**Abstract:**

Colostrum contains macro- and micronutrients necessary to meet the nutritional and energy requirements of the neonatal calf, bioactive components that intervene in several physiological aspects, and cells and microorganisms that modulate the calf’s immune system and gut microbiome. Colostrum is sometimes mistaken as transition milk, which, although more nutritive than whole milk, has a distinct biochemical composition. Furthermore, most research about colostrum quality and colostrum management focuses on the transfer of maternal IgG to the newborn calf. The remaining components of colostrum and transition milk have not received the same attention, despite their importance to the newborn animal. In this narrative review, a large body of literature on the components of bovine colostrum was reviewed. The variability of these components was summarized, emphasizing specific components that warrant deeper exploration. In addition, the effects of each component present in colostrum and transition milk on several key physiological aspects of the newborn calf are discussed.

## 1. Introduction

Colostrum is the first lacteal secretion of mammals after parturition. It is highly energy-dense and contains many bioactive compounds essential for the newborn’s survival and development [1,2]. Colostrum is the ‘starter kit’ for any mammal, providing molecules for all the biological processes the newborn needs to survive now that it is no longer protected by the womb and nourished by the placenta [3,4]. The importance of colostrum is also species-dependent. Due to its physiological characteristics, the female ruminant’s placenta does not allow for the passage of macromolecules to the fetus, which prevents it from acquiring immunoglobulins during gestation, unlike other species such as humans [5,6]. This peculiarity makes it essential for ruminants to consume colostrum immediately after birth, since colostrum has a high concentration in immunoglobulins. In addition to hypogammaglobulinemia, the newborn ruminant also has other physiological constrains, like hypoglycaemia, hypoxia, and hypercapnia, and possible metabolic and respiratory acidosis, the severity of which may depend on the calving difficulty. Fortunately, in addition to immunoglobulins, ruminant colostrum contains macronutrients like proteins, lipids, and carbohydrates, and micronutrients, such as minerals and vitamins, which provide energy and elements for basic cell functions, thermoregulation, and growth [3,7]. Additionally, it contains: (i) many immune factors, antimicrobial peptides, and immune cells that actively protect against specific pathogens and help regulate and “teach” the newborn’s immature immune system [8,9,10]; (ii) endocrine factors that are crucial for the maturation and development of the gastrointestinal tract (GIT) [11]; (iii) signaling molecules that modulate and program gene expression and other cell-related regulatory functions [12,13]; and (iv) microorganisms that help establish the newborn’s microbiota [14]. All these components are required because newborn calves undergo major morphophysiological changes in their first days of life as they adapts to extrauterine life [15], and during this time, they cannot initiate a proper immune response against pathogens [16].

Immunoglobulins, particularly IgG, are the most well-studied bioactive compounds, as colostrum quality is usually defined by its concentration in IgG [17,18,19,20]. However, calves that were fed maternal colostrum instead of colostrum replacer (which has a defined concentration of IgG) showed improved immunocompetence and higher body gains [21], which demonstrates the importance of the whole colostrum matrix for calf wellbeing. The nutritional value and other bioactive components of BC have received less attention from the academic community. Nevertheless, it has been noticed that colostrum contains a wide variety of properties other than the transfer of IgG, which may be just as important. Growth factors are a good example and perhaps the second most studied component of BC. Immune cells have been studied for a long time, and their importance as protectors and immune regulators is a given fact, but it seems that freezing deactivates these cells, which is very problematic as freezing colostrum for later use is a common practice in dairy farms. Some other specific examples of bioactive components that are now receiving attention are the milk fat globule membrane (MFGM), oligosaccharides, lactoferrin, miRNA and nucleic acids, and the BC microbiome. It is known that the most studied components (mainly IgG) vary with season, maternal nutrition, breed, and age, but the variability of other less studied components are not known. These compounds have different roles in protecting and developing the newborn calf, with possible long-term effects. However, there are still questions to be answered, and further research is needed. Colostrum is a unique feed containing hundreds to thousands of different bioactive components [2], which means that there is still so much to learn about colostrum, and with new properties being discovered all the time and with the increasing need for production efficiency, reduced use of antimicrobials, and increased demands for animal welfare, research into colostrum is necessary.

Therefore, in this review, we aimed to provide a comprehensive list of the most studied nutritional and bioactive components in BC. We summarized the range of concentrations for most of the components discussed, distinguishing them from transition and whole milk, and the source of variation of these components was discussed. A characterization of the cellular and microbial populations in colostrum and their interactions with the newborn calf is presented. The role of colostrum components, cells, and microorganisms on selected aspects of neonatal calf physiology was discussed in each section. We also aim to identify compounds that warrant further research, either because they are present in higher concentrations in colostrum than in milk or because there is currently limited information available.

## 2. Bovine Colostrum, Transition Milk, and Inter-Species Variability

Colostrum is a lacteal secretion secreted by mammary epithelial cells prior to parturition, which is physically and chemically distinct from mature milk [22,23]. In ruminants, transition milk is secreted after birth during the first few days of lactation and gradually changes to mature milk in both its physical and chemical proprieties [22]. The definition of the number of days during which transition milk is produced varies between studies, mainly because the transition from colostrum to mature milk varies between cows and the number of milkings and may also be dependent on the starting point, i.e., transitional milk from a cow that produced colostrum with a higher solids content may take more days to become mature milk, compared to a cow that produces colostrum with lower solids. However, it is important to note that lactogenesis II (copious milk production) begins immediately after parturition [23], which means that transition milk secretion theoretically also starts immediately after birth, so the time to first milking may determine whether the fluid milked is more similar to colostrum or transition milk, which may explain some of the variations seen in some colostrum constituents between studies. Nevertheless, in the literature, transition milk can be considered as the lacteal secretion produced during the first and second days after parturition [24], from the second to the fourth [25,26], from the second to the fifth [27], or from the second to the sixth milking [28]. Alternatives to BC are colostrum replacers and colostrum supplements. These products contain around 20% and 50% of fat and protein, respectively, and are highly concentrated in immunoglobulins derived from plasma, serum, or dried colostrum, available in doses containing a concentration of 60 to 200 but usually between 100 and 130 g of IgG, depending as well on if they are used as a replacer or a supplement [29]. However, colostrum replacers are lacking in some bioactive components [30]. Therefore, if good quality colostrum is available, it should not be replaced by colostrum replacers, but it can be provided or added to maternal colostrum if this is not the case or if cows are positive for transmissible diseases such as paratuberculosis (John’s disease). Additionally, it can possibly be used as a nutraceutical, as it has been shown to reduce the likelihood of bovine respiratory disease outbreaks in calves aged 14 to 50 days [31].

BC is the most studied prepartum lacteal secretion in domestic animals, while Human colostrum is perhaps the most studied. Colostrum from the human species, like many other mammals, is characterized by having a higher concentration in IgA, while colostrum from ungulates is more concentrated in IgG. The mechanisms that lead to the transfer of components to colostrum are species-specific [2]. Nevertheless, in all mammal species, colostrum is important for systemic and enteric immunization, especially in species where placental passive immunization does not occur, as in the case of ungulates. Within ruminant farm species, macronutrients and IgG concentrations vary considerably. The concentration of fat (4.0–8.0%), protein (10.5–17.1%) and lactose (3.2–4.4%) in goat colostrum has been shown to vary between breeds [32]. According to these results, the variation in fat and protein seems to be similar between goats and dairy cows, but lactose may be higher in goat colostrum (macronutrient concentrations in BC are discussed in the following chapter). However, studies with Majorera goats showed different results, with colostrum higher in fat, lower in protein, and similar in lactose concentration to that of dairy cows [33,34,35]. Ewe colostrum from different breeds was higher in fat (4.0–13.7%) and protein (13.8–22.5%) concentrations and similar in lactose (2.4–3.8%) concentration to dairy cow colostrum [32,36]. Colostrum from Santa Inês sheep had concentrations of fat and lactose normally higher than colostrum from dairy cows but lower in protein concentration [37]. There is a marked variation in IgG concentration in colostrum [2], and the average values from these three species are similar but they fall in different ranges across studies [32,34]. With regard to other components, there is not yet enough data to make a proper comparison, but there is evidence of differences in minor components [34]. Therefore, there is no specific rule for colostrum components between these three species, although some tendencies can be assumed. However, it is reasonable to assume that sheep colostrum has the highest solids content compared to cow and goat colostrum [34,38] and that colostrum components may vary between breeds [32]. 

Moreover, the importance of colostrum, mainly BC, for farm animals is gaining popularity as a food ingredient [39] and as a nutraceutical for humans [40,41]. It is also being explored for potential applications in anti-tumor therapeutics for specific cancers [42]. Due to its nutritional value and low availability, BC has a high market value compared to other by-products of the sector [43], and its demand is projected to increase [44]. This heightened market value accentuates the significance of conducting research to enhance both the availability and quality of bovine colostrum (BC), both on the farm and throughout the processing stages.

## 3. Macro- and Micronutrients in Bovine Colostrum

Colostrum contains water, proteins, fats, carbohydrates (macronutrients), minerals and vitamins (micronutrients), as well as bioactive components such as immunoglobulins, hormones, enzyme inhibitors, growth factors, and nucleic acids, albeit in smaller quantities. Some of these components can be described as having both nutritional and bioactive roles; however, despite their nutritional value, the importance of specific molecules goes beyond straightforward caloric content, which is why they will be considered as bioactive molecules in this review. 

All nutrient and non-nutrient factors except water make up the total solids (TS) of colostrum, which is officially measured using the air-oven loss-on-drying technique (Method 925.23 of the AOAC). TS in colostrum reflect its overall quality and can be measured on the farm level with a with a Brix % refractometer, which also correlates well with protein concentration [45,46]. Another instrument is the colostrometer, which measures specific gravity, relating to total protein and immunoglobulin content in colostrum [47]. Dry matter, protein, fat, and lactose analysis have official methods. Still, in most recent studies spanning approximately 25 years, these components have been analyzed using an automated mid-infrared method, employing Milkoscan equipment. Several peripartum dam-related factors significantly influence the yield of nutrient and non-nutrient components in colostrum. However, more knowledge is still needed to understand how these factors affect the production and transfer of these components other than IgG [48]. Factors such as farm size, prepartum feed, breed, parity, season, calving month, udder health, time interval from calving to milking, colostrum weight, and method of analysis can contribute to IgG variation [49,50,51,52]. Some of these factors also affect the nutrient component of colostrum, but there are still very few studies regarding this subject [53]. Table 1 and Supplementary Figure S1 show the variability of the main components in BC. The coefficient of variation was calculated to highlight the variability of each component across the literature. The authors tried to provide a broad range of studies across different regions, but the majority are concentrated in the subcontinent of North America. Specific information on each component is provided in the respective subchapters.

### 3.1. Macronutrients

#### 3.1.1. Total Solids

During the first few weeks of a calf’s life, colostrum and milk are the primary sources of water. Voluntary intake of free water is low during this period and gradually increases with age, weight, and dry matter intake [70,71]. The quality of colostrum is determined by the proportion of its non-water constituents, commonly referred to as TS. Most review articles on colostrum composition report the same mean value of 23.9% for the TS content of BC [4,22,72], all of which (indirectly) trace back to a paper published in 1950 with Holstein cows [54]. However, more recent research should be considered when considering an overall value for BC. More recent papers also report a percentage of total solids similar to Parrish et al. [54]; for example, a study with Jersey cows in the United States (n = 96; 23.6%) [18], another with dairy cows from Switzerland (n = 24; 24%) [73], and another with Egyptian Holstein cows (n = 12; 24.2%) [55]. A more extensive study (n = 496) showed a higher variation, including differences between different regions of the United States [61]. The mean values ranged from 20.6 to 24.1%, the minimum reported value was as low as 1.7%, and the maximum reached 33.1%. In a study in Pennsylvania (n = 55), the reported mean TS (27.6 ± 5.8%) was higher than in the aforementioned studies [56]. Two other studies reported similar values in Iran with 365 Holstein cows [52] and in India with Jersey cows [62]. Jersey cows are known for their ability to produce milk with a higher concentration of solids than Holstein cows [74]. However, Morrill et al. [61] found no differences in the total solids of colostrum between Holstein (n = 494) and Jersey cows (n = 87). In contrast, a study in Brazil with Jersey cows reported higher values of TS when compared to other studies (31.3%) [75]. Local dairy breeds may have lower TS than Jersey and Holstein cows [62,76], but there are very few studies evaluating the colostrum composition of local dairy breeds to support this statement. There is still uncertainty regarding whether the reduced solids content in indigenous breeds has a similar impact on their offspring as observed in calves of Holstein or Jersey breeds. Further exploration is necessary to consider other factors, such as calf size, which directly affects their dietary needs, and any regional discrepancies across countries. Another interesting factor is related to the number of parities. In some studies, colostrum from first and second lactation cows was of lower quality [19,77], which was somewhat expected, as older cows tend to have more antibodies than younger cows. However, other studies have shown that colostrum from first lactation cows did not differ from that of third lactation cows and that colostrum from second lactation cows had lower TS [53,59] and IgG [18,49]. No explanation has yet been found for these results.

These studies indicate a degree of homogeneity in the mean TS concentration of colostrum within the herd, with some possible variations between regions and breeds, as seen in Table 1. From these studies, a mean concentration of total solids of 24.7% can be inferred. This is slightly higher than the previously reported value (23.9%) and above the limit for good quality colostrum (22%), which should estimate an IgG concentration of 50 mg/mL. However, the minimum values are represented by colostrum of lower quality (<22%), so it is still important to regularly assess colostrum quality [4]. This variation can compromise the calves’ nutritional requirements and passive immune transfer if colostrum quality is not assessed prior to feeding or if the calf suckles directly from the dam [78].

#### 3.1.2. Proteins

Protein is the most abundant nutrient in colostrum and can be four times higher than in milk [72]. In BC, it is found at a concentration of 15.5%, ranging from 2.6 to 31.6% (Table 1).

Breed can affect the protein percentage in colostrum [62], and dairy cows with lower milk yield tend to have higher milk solids, yet Morrill et al. [61] found no differences in colostrum protein content between Holstein and Jersey cows. The protein percentage of BC was lower in first and second parity cows than in cows with three or more parities [66]. Zarei et al. [52] and Soufleri et al. [53] found significantly higher values only in cows with four or more parities. At the same time, Morril et al. [61] found no differences in the protein percentage between parities. It would be interesting to investigate the variation in protein fractions in the colostrum from cows with different parities, as the lower protein concentrations in younger cows could be attributed to lower immunoglobulin concentration; however, as previously mentioned, this may not always be the case, at least for cows in first, second, and third parity. A higher protein percentage in colostrum was recorded during autumn and winter in Northern Greece [53] and during winter in Northern Ireland [66]. In contrast, a study conducted in Egypt found no differences in colostrum protein content between seasons [52]. Nevertheless, it was observed that protein percentage was lower under heat stress conditions compared to thermoneutral conditions [64]. To better understand these differences, controlled experiments with consistent climatic conditions could help clarify whether the observed differences are due to environmental factors, such as temperature and photoperiod, or to confounding effects. There appears to be no marked effect of dry period length on protein percentage in BC [53], but a higher protein percentage was observed in colostrum from cows with a very long dry period (≥16 weeks) [66]. Cows receiving bovine rhinotracheitis vaccinations produced 11 g/L more protein in colostrum than unvaccinated cows, probably due to the increase in immunoglobulins, since it was significantly higher in vaccinated compared to unvaccinated cows [66]. Colostrum sampled from cows vaccinated against neonatal calf diarrhea also showed increased Brix values [79]. 

Colostrum has a higher concentration of caseins (insoluble fraction) and whey proteins (soluble fraction) than milk [22]. Casein is present in the form of colloidal casein micelles (CMs); these CMs are larger in colostrum than in milk [80]. CM size is important in milk processing, but the role it plays for the newborn calf is unknown. Compared to milk, BC has a higher percentage of κ-casein, a lower percentage of α_s_-casein, and a similar percentage of β-casein [81]. Casein is involved in the transport of minerals and trace elements [72,82], and it can also reduce proteolytic degradation, acting as an enzyme-inhibiting protein, preserving intestinal integrity and function (protecting epidermal growth factor from digestion), and aiding in the activity of other biologically active peptides [83,84].

The whey fraction, in addition to its nutritional value, is composed of a large number of different proteins that play an important role in several biological processes [85], of which the most studied in milk are: α-lactalbumin, β-lactoglobulin, bovine serum albumin (BSA), immunoglobulins (IgG, IgM, IgA, IgE, and IgD), bovine lactoferrin, and lactoperoxidase [86]. According to our research, their concentration in bovine colostrum varies widely (Table 2 and Supplementary Figures S2 and S3). Some proteins such as casein, α-lactalbumin, and β-lactoglobulin are synthesized in the mammary gland, while others such as immunoglobulins, BSA, and lactoferrin are actively transported from the blood into the lumen of the mammary gland [17]. The importance of these proteins for the human species is well-established (Madureira et al., 2007), but their importance for newborn ruminants is not as well known, except for immunoglobulins. Colostrum proteins are a source of amino acids that the newborn calf can use for protein synthesis. However, the newborn gastrointestinal tract (GIT) exhibits low proteolytic activity, and colostrum, despite containing trypsin inhibitors [87], still allows for some exogenous protein hydrolysis alongside intracellular protein degradation, which contributes to amino acid supply [88]. Whilst some proteins, such as α-lactalbumin, can be rapidly hydrolyzed to amino acids in the abomasum, caseins form a clot, which undergoes slower degradation, thus providing a more spaced source of amino acids [3,88]. Curd formation retains caseins and lipids in the abomasum and slowly releases the whey fraction into the gut, where it can be effectively absorbed. This may explain why calves without curd formation had lower serum IgG and IgA levels [89].

Immunoglobulins and β-lactoglobulin are proteins that are highly resistant to degradation [88]. Compared to milk, colostrum has a higher β-lactoglobulin/α-lactalbumin ratio, suggesting that β-lactoglobulin may play a specific role for the newborn [90]. Β-lactoglobulin is involved in the digestion of milk lipids by improving the activity of pregastric lipases [91], enhancing the digestion of colostrum fat, promptly providing energy to the newborn. β-lactoglobulin also functions as a transporter of small hydrophobic ligands, such as retinol, cholesterol, and vitamin D, to specific intestinal receptors [86]. α-Lactalbumin is present in the bovine colostrum and milk at lower concentrations than in human colostrum and milk, playing a role in the synthesis of lactose [92]. BSA helps maintain oncotic pressure and is involved in the binding and transporting of molecules such as fatty acids, bilirubin, hormones, and minerals [93]. It is transferred from blood to colostrum and is usually considered an indicator of the permeability of the blood–milk barrier [19,94,95]. It has been reported that a higher proportion of BSA in colostrum can affect the transfer of immunoglobulins from the intestinal lumen of the calf to the circulatory system due to the limited capacity of the macromolecular transport mechanism [96].

**Table 2:** Concentrations of the major proteins in bovine colostrum.

Protein	Concentration	Reference
Caseins (αs, β, к) (mg/mL)	48–96	[22,82,97,98,99]
α-lactalbumin (mg/mL)	2.0–7.5	[94,98,99,100]
β-lactoglobulin (mg/mL)	4.8–24	[94,98,99,100]
Bovine serum albumin (mg/mL)	0.45–2.5	[19,94,101]
Immunoglobulins (IgG, IgA, IgM) (mg/mL)	47–106	[19,56,102]
Lactoferrin (µg/mL)	34–4400	[56,97,99,100,102,103,104,105]
Transferrin (µg/mL)	187–1070	[103,104,105,106]
Lysozyme (µg/mL)	0.4–1262	[100,102,107]
Lactoperoxidase (µg/mL)	22.8–22.8	[102]

Values represent the range of mean concentrations.

#### 3.1.3. Lipids

Lipids are the second most important macronutrients of colostrum after proteins and are the main source of energy that the calf receives at birth. It is also the component with the highest variability, ranging from almost 0 to around 26%. The average fat percentage in BC is around 5.5% (Table 1). Breed can affect the fat percentage in colostrum [62], and dairy cows with lower milk production capacity tend to have higher milk solids, but Morrill et al. [61] found no differences in colostrum fat content between Holstein and Jersey cows. The fat percentage of BC is higher in primiparous cows than in multiparous cows [52,53,61,66]. This can be explained by the generally lower yields of primiparous animals; however, protein concentration is generally lower in primiparous cows. Season may have a significant effect on fat percentage, but this is likely to be region-dependent. In Northern Ireland [66] and Northern Greece [53], colostrum fat percentage was higher in spring. In Egypt, there were no differences between seasons [52]. Nevertheless, it was observed that fat percentage was lower under heat stress conditions compared to thermoneutral conditions [64], which is usually associated with lower dry matter intake. Without climatic information, comparing seasons across different studies and regions could be misleading, and other factors that are probably indirectly related, like feed availability, could be influencing these results. There is not much information on the effect of dry period length on fat percentage in BC, but it seems that a dry period length of more than 60 days may result in colostrum with a higher fat content [53,66], which could be explained by increased mobilization of the body’s energy reserves associated with increased body condition during a prolonged dry period [53]. In addition, a significant (*p* = 0.001) positive association was observed between fat percentage in BC and feeding straw and grass silage 7 to 9 weeks before calving and vaccination against leptospirosis [66]. The impact of diet on colostrum fat concentration needs to be better understood, especially as the onset of colostrogenesis is not well understood. Thus, dietary differences between the dry period and the peripartum period may influence colostrum composition. Nevertheless, feeding forages increase ruminal acetic–acid formation, which influence the synthesis of lipids in colostrum and milk [108].

The main classes in the lipid fraction are triglycerides or triacylglycerols (TAG; the main class within the lipid fraction), phospholipids (the two main classes are glycerophospholipids and sphingolipids), free fatty acids (FA; short- and long-chain, saturated and unsaturated), eicosanoids, and sterols (the most abundant of which is cholesterol) [7,109]. Lipids are an energy source, serve as structural components of membranes, are precursors for other molecules, and act as actuators in various biological processes [110]. The lipids present in colostrum and milk are almost entirely in the form of milk fat globules (MFGs), a lipid droplet containing mostly TAG that is formed in the endoplasmic reticulum of the alveolar epithelial cells of the mammary gland [111]. MFGs are then secreted by fusion with the plasma membrane of the alveolar epithelial cell, acquiring a peripheral bilayer called the milk fat globule membrane (MFGM), which contains polar lipids, cholesterol, glycoproteins, gangliosides, and enzymes in its structure [112,113]. The functions and mechanisms of action of MFG and MFGM are not fully understood, but they may contribute to antimicrobial effects, gut maturation, structural development, and the establishment of early neonatal gut microbiota [112,114]. 

Lipids also act as a source of energy, with FA oxidation providing energy to sustain gluconeogenesis [3]. This source of energy is particularly important in neonates, as lactose intake from colostrum is insufficient to meet glucose requirements [30]. In fact, a high postanal capacity to oxidize FA has been reported in several species [115]. FA oxidation capacity increases during the first 24 h after birth, especially medium-chain FA [15]. Most of the energy comes from TAG, since they are the major constituents in milk fat, as around 95–98% of the bovine milk fat are TAG [116]. Gastric and pancreatic lipases hydrolyze TAG to FA and monoglycerides, which are absorbed in the intestine. These lipids then undergo several processes of re-esterification and hydrolysis until they reach the endothelial cells, where they can serve as a substrate for bioactive molecules, be oxidized to provide immediate energy, or be resynthesized into TAG, where they are transported to extrahepatic tissues such as muscle and adipose tissue for storage and later use as a source of FA and energy [110,115,117].

The energy provided with FA oxidation can also be used as a heat source, as newborns are born poorly insulated and have low metabolic rates [3,4,48]. Rectal temperature drops by an average of 0.5 °C in the first 90 min, mainly due to the wet hair coat, which has a low thermal insulation, and due to the evaporation of amniotic fluid, which removes heat from the surface of the skin [118]. Other factors like wind velocity can also increase the rate of heat loss. Thus, heat production in the first hours of life is not enough to meet heat losses at 20 °C [118]. The newborn calf is born hypoglycaemic and has very limited energy reserves, as only 3% or less of its body weight is made up of lipids, most of which are structural [61]. It is speculated that the endogenous lipid content of a 40 kg newborn calf could support summit metabolism for 15 h and that glycogen reserves would be depleted in ≤3 h [119]. In cold environments where hypothermia may occur or in the event of dystocia, the situation may be exacerbated [118]. Nevertheless, the lower critical temperature for a newborn calf is close to 22 °C. Rectal temperature may rise around 15 h after birth due to more efficient peripheral vasoconstriction and the increased insulating capacity of the now dried hair coat [118], although it would depend on the environmental climatic conditions and housing characteristics. Approximately 1.5% of calf BW is brown adipose tissue (BAT), mainly located in the perineal, prescapular, pericardial, and abdominal regions [120]. BAT contributes to non-shivering thermogenesis and is mediated by the cold-induced secretion of norepinephrine by the sympathetic nervous system, which stimulates the expression of the UCP1 protein. UCP1 uncouples electron transfer from ATP synthesis in the mitochondria of brown adipocytes, so the energy derived from FA oxidation is dissipated as heat instead [121]. This strategy allows hypothermia to be avoided until nursing occurs. However, some gestational factors can affect the amount of BAT that the newborn has [121]. In addition, BAT is gradually replaced by white adipose tissue during the first few days of life [120]. Therefore, the high caloric content of colostrum makes it a good source of energy for heat production [122]. The energy content of BC can vary substantially, considering the variability in the nutrient composition of colostrum (Table 1). Reported average values for the metabolizable energy (ME) of BC range from 24 to 25.6 MJ/kg DM, however, when considering extreme values, can range from 14.2 to 34.8 MJ/kg DM [53,56,123,124]. As expected, it is higher than the ME of whole milk, which is around 18.1 to 22.5 MJ/kg DM [123,124,125]; however, colostrum on the lower end can have lower energy than whole milk. According to the NRC, a calf with a body weight of 40 kg has a maintenance ME requirement of 6.7 MJ/day [123]. Considering an average value of 24.8 MJ/kg DM in colostrum and the energy requirements of a 40 kg calf (6.7 MJ/day), this calf would need 0.27 kg of DM as colostrum per day. Considering a 24.7% of DM in colostrum, this calf would need to consume a total of 1.1 L of colostrum to meet its ME requirements. This value is way below the current recommendations of 10% of the calf’s body weight, which in this case would be 4 L of colostrum (26.8 MJ of ME). This recommendation is based on immunological requirements (mostly IgG) and not on energy requirements. The rest of the energy would then be used for thermogenesis (heat production) and growth (protein turnover), both of which are demanding activities in early life. Therefore, the energy provided by colostrum is essential soon after birth to maintain normal functions, including normothermia. 

#### 3.1.4. Carbohydrates

Colostrum contains carbohydrates in the form of lactose, oligosaccharides, glycoproteins, glycolipids, and nucleotide sugars. Lactose is the major carbohydrate in colostrum, with an average concentration of 2.8%, ranging from less than 1% to 5.2% (Table 1). Nazir et al. [62] observed that local dairy cows had lower lactose in colostrum than Jersey cows, and Zarcula et al. [126] observed lower lactose levels in Romanian Black and White cows compared to Holstein–Friesian cows. Morrill et al. [61] found no differences in colostrum lactose content between Holstein and Jersey cows. The effect of parity on lactose content in colostrum is not marked when compared to protein or fat. However, there is a tendency to decrease when the number of parities increases [52,53,66], though this number varies between studies. In a study in Northern Ireland [66] and in Egypt [52], lactose percentage was higher in autumn but lower in a study in Northern Greece [53]. Nevertheless, lactose percentage was observed to be lower under heat stress conditions compared to thermoneutral conditions [64]. As colostrum yield increased, lactose percentage also increased, but protein percentage decreased [53]. Lower lactose percentage was also observed in colostrum from cows with a very long dry period (≥16 weeks) but not in cows with a shorter dry period [66]. The effect of dry period length on colostrum composition is not entirely understood. The majority of studies focus on IgG levels and in shorter or no dry periods at all, but even these results are not consistent [127,128,129]. It would be interesting to understand the effect of longer dry periods on colostrum composition, despite the economic constraints associated with longer periods. 

The concentration of lactose is lower in colostrum than in milk [7,54], and as it has osmoregulatory functions, a high concentration of lactose would increase the movement of water from the cytoplasm of the mammary epithelium into the secretory vesicles and subsequently into the colostrum [7]. This would reduce its overall quality, since it would have fewer components per litter. Therefore, lactose concentration in colostrum increases with the time until colostrum collection, but the concentration of other components decreases [53,66]. This can explain why older cows tend to have lower lactose levels in colostrum than younger cows, since TS and protein levels usually increase with the parity number. The low levels of lactose in BC make lipids the main source of energy available to the newborn calf in its first feed. However, lactose in colostrum is necessary to increase the water content of colostrum during the final phase of prepartum, otherwise the colostrum would be too dense for the calf to suckle [2].

Lactose is a disaccharide that, when hydrolyzed, provides glucose and galactose as energy sources [130]. Carbohydrates, mainly glucose and lactate, are the fetus’ main form of constant energy supply, which changes after birth to a high-fat inconstant energy supply [15]. Calves are born hypoglycaemic, and lactose intake from colostrum does not fulfil the calf’s glucose requirements [30]. Therefore, calves must rapidly establish endogenous glucose production (glycogenolysis and gluconeogenesis processes) [3,30]. During the first hours of life, the calf can use the hepatic glycogen stored during the last phase of gestation to maintain normal blood glucose levels [30]. It has also been shown that colostrum feeding improves glucose absorption [131]. However, prolonged absence of feeding can lead to hypoglycaemia when there is no exogenous glucose intake, the glycogen stores are depleted, and gluconeogenesis still needs maturation [30,131,132]. Lactate (and to a limited extent, glycerol) is the main substrate for gluconeogenesis in the early stages of life, and as the rumen develops, propionate becomes the main substrate as the production of volatile fatty acids increases [15]. 

### 3.2. Micronutrients

#### 3.2.1. Minerals

There is a large variation in the total mineral concentration of BC, which can vary with the time interval between parturition and milking [56,133,134], the milking strategy [26], and the number of milkings [134,135]. Unlike most colostrum constituents, mineral content does not seem to be negatively associated with colostrum yield [135]; however, further research is needed in this area, as well as with other variation factors. The most abundant minerals in colostrum are Ca, P, K, Na, and Mg, while Zn, Fe, Cu, and Mn are present in lesser amounts. Colostrum has a high concentration of Ca, P, Na, Mg, Fe, Se, Cu, and Zn compared to milk but a lower concentration of K and Mn [72,133,135,136]. Some minerals, such as Ca, P, and Mg, seem to be in higher concentration in colostrum from first or second parity cows compared to cows with three or more parities [134,135]. 

Each mineral has different physiological functions in the organism, and therefore, adequate levels of macro- and microminerals are essential for neonatal health and growth. However, the role of colostrum minerals in newborn calves is not fully understood, and there is some disagreement as to whether maternal mineral supplementation improves colostrum quality [137]. In a recent study, both cows and calves showed increased levels of blood IgA, IgM, and total antioxidant capacity, following prepartum supplementation with Mn, Zn, and Cu, but results were dependent on the source of supplementation [138]; protein, fat, and lactose in colostrum did not change with treatment, and immunoglobulins were not measured in colostrum. Ca is essential for skeletal development, and absorption rates can reach 99% in young calves (10 days), decreasing with age to 22% in adult cattle [139]. Mineral supplementation over dietary requirements did not improve colostrum mineral concentrations in Brahman cattle [133] or sows [140], except for selenium. The efficiency of absorption is likely to decrease when dietary minerals, such as Ca, are supplemented above requirements [133,135], which also depends on the source of the mineral, as in the case of selenium [141]. More recently, it has been shown that pre-natal mineral and vitamin supplementation can alter the newborn calf microbiome at different sites of the body, affecting early microbial colonization [142]. Research into the mineral composition of colostrum is limited, and therefore, very few conclusions can be drawn.

#### 3.2.2. Vitamins

Vitamins can also be included as bioactive components, but for the purposes of this review, they will be referred to as micronutrients. Colostrum is an important source of vitamins essential for the health and growth of the newborn calf, and delayed colostrum intake can impair cell growth and differentiation and increase susceptibility to infectious diseases [4,73]. Fat-soluble vitamins (vitamins A, E, and D) are present in higher concentrations in colostrum than in milk, but water-soluble vitamins (vitamins C and B complex) do not follow the same trend [7,22,56,143,144,145,146,147,148]. Compared to milk, colostrum has only slightly higher concentrations of vitamin C, and within the B complex, vitamins B1, B2, B6, B9, and B12 may be more concentrated in colostrum, B5 and B7 are usually less concentrated, and B3 seems to be as concentrated as in milk. [7,72]. Nevertheless, vitamin C is the most concentrated vitamin in BC. Table 3 shows the concentrations of vitamins in BC, as well as their physiological roles in cattle. There is a lack of recent research on the vitamin content of BC. As genetics and feeding strategies have changed in recent decades, updating these values may be relevant. Considering the available results, the variation in vitamin concentrations in colostrum appears to be more related to analytical difficulties rather than variable secretion patterns [72]. The factors influencing vitamin variation in BC are poorly understood but are known to be influenced by the time between parturition and milking and by the prepartum diet [7,149]. Heat treatment of colostrum (60 °C for 60 min) does not appear to affect levels of vitamin A, vitamin E, or β-carotene [150]. More research is required to comprehend the factors influencing the vitamin content of colostrum. Vitamin deficiencies can notably impact calf health, and since colostrum provides a substantial amount of these nutrients, further investigation is warranted.

Vitamin A is important for protection against infection, immune function, cell growth and differentiation, maintenance of epithelial surfaces, and vision [151]. However, calves are born deficient in vitamin A and β-carotene; supplementing cows with vitamin A during the dry period can increase plasma retinol concentrations in calves [152]. Vitamin E appears to be transferred into colostrum by a mechanism involving low density proteins [7,153]; it acts in the lipid phase as a radical scavenger, protecting phospholipid membranes from peroxidative damage, and increases the functional activity of neutrophils [154]. Vitamin B2 is found in higher concentrations in colostrum compared to other B vitamins. The concentration of B2 in colostrum exceeds the requirement of 100 µg/100 g of dry matter consumed by pre-weaned calves [155], suggesting a specific role for the newborn calf. To the authors’ knowledge, its specific role in the newborn calf has not been studied, but a deficiency of B2 results in mucosal lesions and growth-related problems in young calves [156]. Calves can be born with vitamin D deficiency and remain at low levels throughout the pre-weaning period, which has been shown to affect the immune system [148,157]. Cows are able to synthesize vitamin C primarily in the liver [158]. However, calves do not begin to synthesize endogenous vitamin C until about 2–3 weeks of age, making them dependent on the vitamin C provided by milk [159]. Cases of scurvy (a sign of vitamin C deficiency) have been reported in calves [160]. Non-colostrum-fed calves with a plasma vitamin C concentration of less than 0.15 mg/dL showed active infections and swollen navels, whereas injections of 500 mg/dL alleviated the symptoms [159]. 

## 4. Bioactive Components in Bovine Colostrum

Bioactive components (that can also be referred to as nutraceuticals) are natural essential and non-essential molecules that act in the animal organism, usually with health benefits beyond basic nutritional value [161]. Compounds such as vitamins, hormones, growth factors, certain proteins, carbohydrates, and lipids, as well as molecules such as nucleotides, polyamines, and miRNA have been identified as bioactive components in colostrum [11,13,48,55,97,162,163,164,165]. These substances can be either of blood origin or produced in the lactocytes of the mammary gland [162].

Bioactive components from colostrum stimulate the development and function of the GIT, modulate the GIT microbiota, and provide local protection. There are multiple receptors for bioactive components in the GIT that trigger multiple events, making it difficult to explain the function of a specific factor [166]. Although there are many bioactive components in colostrum [2], only a few have been the target of research. Therefore, only the currently most relevant bioactive components will be reviewed.

### 4.1. Bioactive Proteins

In addition to being a source of amino acids, the proteins in colostrum can perform several functions for the newborn. These proteins are found in the whey fraction, and some of them have already been mentioned above. These bioactive proteins act through a variety of mechanisms, but their role is largely related to host defence. 

#### 4.1.1. Immunoglobulins

Colostral immunoglobulins are responsible for protecting the immunological naïve newborn calf against pathogens by activating and regulating the innate immune system. Although there is still much to learn, they have been extensively studied and reviewed in other papers, so only a brief mention of their transport mechanisms and concentrations in colostrum follows.

Immunoglobulins are a family of high molecular weight proteins with similar physicochemical properties and antigenic determinants [167]. The main immunoglobulin classes present in bovine colostrum are IgG, IgA, and IgM [17], with IgD and IgE also present [4]. IgG is the predominant immunoglobulin in bovine colostrum, whereas IgA is predominant in primate colostrum [23]. IgG represents over 50% of the total protein of BC [168]. IgG is divided into three subtypes: IgG1, IgG2, and IgG3 [168]. IgG1 predominates over IgG2 in bovine colostrum by approximately 7:1, although blood concentrations are similar [169]. Nevertheless, IgG1 in blood decreases during colostrogenesis, which may be due to the passage of IgG1 from blood into colostrum [2]. IgG3 is present at even lower levels and has only recently been detected in BC [168].

IgG in colostrum is found in concentrations ranging from 0.68 to 216.70 mg/mL; IgA concentrations range from 0.13 to 22.14 mg/mL and IgM from 0.18 to 14.01 mg/mL [19]. As IgG is the predominant immunoglobulin in BC, only this class is considered in quality assessments, and a colostrum with 50 mg/mL of IgG is considered good quality colostrum [4]. However, for effective immunization, factors such as the amount of colostrum ingested, the time between birth and ingestion, microbiological contamination, and the method of administration must be taken into account [4].

IgG1 is selectively transported from the bloodstream into colostrum by specific receptors on mammary alveolar epithelial cells, a pH-dependent process called transcytosis [4,23], while IgG2 (and BSA) are recycled [170]. IgG1 and IgG2 differ in their amino acid structure, which may explain their different transport mechanisms [170]. IgA and IgM are produced in smaller quantities by plasma cells in the mammary gland [17]. While immunoglobulin transport appears to be selective at the mammary gland, the intestinal tract of the calf is non-specific for any immunoglobulin class until 24 h after birth [17]. Immunoglobulins and complement proteins are resistant to gastric acids [3,72], which can increase their bioactive functionality along the GIT or allow for absorption without compromising their structural integrity [112]. IgG1 can also be re-secreted into the GIT via FcRn receptors, providing local specific immunity [29,171].

#### 4.1.2. Lactoferrin and Transferrin

Lactoferrin (LF), a member of the transferrin protein family, is an iron-binding glycoprotein synthesized in the mammary gland and in other exocrine glands and is therefore present in colostrum, milk, saliva, and bronchial, cervicovaginal, and gastrointestinal fluids [72,84,172,173]. LF is not present in the lacteal secretions of all mammalian species; for example, it has not been detected in dogs, rats, and rabbits, but it is one of the most abundant glycoproteins in ruminant and human milk, although human milk is much more concentrated in LF than bovine milk (100 times or more) [173,174]. In contrast, rat and rabbit milks are more concentrated in transferrin than human milk, in which it is undetectable [175]. In cattle, both LF and transferrin are found in higher concentrations in colostrum than in milk [103], and transferrin is higher in blood [106]. The mammary gland secretes a high mass of LF during the dry period and colostrogenesis, and it may influence the release of IgG1 in colostrum by increasing intracellular pH, facilitating the release of IgG1 from FcRn, since this receptor depends on a pH < 6.5 or >6.5 to either bind or release IgG1 [2]. The concentrations of LF and transferrin in bovine colostrum are shown in Table 2 and Supplementary Figure S3.

LF has been described as having many functions and may be relevant to GIT growth regulation in neonates [84]. Some functions are the ability to regulate iron absorption [176,177]; improvement in the intestinal epithelial barrier by promoting cell growth, decreasing paracellular permeability, and increasing alkaline phosphatase activity and transepithelial electrical resistance [178]; ability to be released from plasma neutrophils during infection or inflammation, possibly contributing to the activation of other immune cells, thus providing protection against pathogens [179,180]. However, it can also regulate the inflammatory process by inhibiting the progressive inflammatory cascade [72]. 

It appears that LF plays a greater role as a protective mechanism against infection in the dry mammary gland than in the lactating mammary gland, probably because of the low concentration of LF and the high concentration of citrate in the latter stage [180,181]. However, when LF is saturated with iron, it loses its bacteriostatic effect [182], and given that citrate is an iron chelator, it seems that the low concentration of LF in the lactating mammary gland may be the main reason for the reduced antimicrobial effect of LF at this stage. Nevertheless, LF has a 300-fold higher affinity for iron than transferrin, which may not be relevant at physiological pH but allows LF to retain its iron-binding capacity in a more acidic environment, particularly in the presence of citrate [177]. LF has received increasing attention as a multifunctional protein, but its mechanisms are not yet fully understood.

#### 4.1.3. Proline-Rich Polypeptide 

Proline-rich polypeptide (PRP), also known as colostrinin, is a complex of at least 32 peptides present in colostrum of various species, including bovine [183,184]. PRP is probably derived from the partial proteolysis of annexin and β-casein [184,185]. The therapeutic effects of PRP have been studied in laboratory animals and in humans, particularly in patients suffering from Alzheimer’s disease [185,186]. PRP can help prevent oxidative damage [187], which is important for newborn calves, as they are susceptible to oxidative stress in the early stages of life [188]. It has also been shown to be effective in improving long-term memory in newborn chickens [189] and in modulating adaptive and innate immunity [190,191]. Immunocompromised rats infected with enterotoxigenic *E. coli* had reduced endotoxin levels and infected lymph nodes when treated with PRP [192]. PRP helps regulate cytokine production and has been shown to reduce allergic inflammation in murine [193]. These antiallergic properties could be helpful for calves fed milk replacers, since allergic reactions in calves to soybean flour in milk replacers have been reported [194]. 

#### 4.1.4. Enzymes

Colostrum, like milk, contains many enzymes that perform functions associated with the host defence mechanism against microorganisms and oxidative damage, as well as with many essential metabolic processes such as catalysis, lipolysis, and proteolysis.

Lactoperoxidase (LPO) is one of the most frequently mentioned enzymes in the literature. It is a glycoprotein, a member of the family of haem peroxidase enzymes, secreted by the mammary gland into colostrum [84,184]. The main biological function of LPO is to defend against microorganisms by generating reactive oxygen species (ROS), which is effective against a wide range of bacteria but also has antiviral and tumoricidal activities [180]. This enzyme has been shown to be very resistant to proteolysis, highlighting its importance in the defence of the calf’s GIT [180]. LPO and LF in milk lose activity during heat treatment above 70 °C for 30 min [195]. In this study, where different temperatures were tested, raw milk samples showed a lower growth rate per hour than treated milk of *Streptococcus thermophilus*, *Lactococcus lactis*, *Pseudomonas fluorescens*, and *Escherichia coli* in whey because as temperature increased, the total protein content in whey decreased significantly, which also reduced the bacteriostatic activity in milk.

Lysozyme is an enzyme present in colostrum and milk [100,102,107] with specific hydrolytic activity against the peptidoglycan in cell walls of Gram-positive and Gram-negative bacteria [196]. It is more effective against Gram-positive bacteria because their cell walls contain up to 90% peptidoglycan [197]. Compared to humans or other species, such as horses, this enzyme is present in lower concentrations in bovine milk, probably too low to contribute effectively to the overall bacteriostatic and bactericidal activity [180,197]. Reported concentrations of lysozyme in bovine colostrum vary widely and are usually lower than other enzymes with antimicrobial activity (Table 2), but it generally increases after the first milking [198]. 

Other enzymes present in bovine colostrum include catalase, superoxide dismutase, and glutathione peroxidase, which have antioxidant properties, and β-1,4-galactosyltransferase, lactate dehydrogenase, alkaline phosphatase, and gamma-glutamyltransferase, which catalyze important biological reactions, and esterases, lipases (such as lipoprotein lipase), proteases (such as tissue plasminogen activator), and ribonucleases (such as ribonuclease II-1), as well as enzyme inhibitors, which are present in very high concentrations but rapidly decrease with time after birth [7,11,154,166,197,199,200,201]. 

#### 4.1.5. Cytokines

Cytokines in colostrum are divided into interleukins, interferons, and tumor necrosis factors, which are responsible for modulating the immune system [7]. Some of these pro-inflammatory cytokines, such as IL-1β, IL-6, and tumor necrosis factor-α (TNF-α), and acute phase proteins, such as serum amyloid A and haptoglobin, can influence the concentration of these molecules in the serum of calves during their first weeks of life [202]. Weaned pigs supplemented with colostrum also showed changes in cytokine mRNA expression in spleen- and gut-associated lymphoid tissues, with increased expression of IL-2, IL-4, IL-10, IL-12 and decreased expression of IFN-γ [203]. IL-1Ra, IL-1β, IL-6, TNF-α, and IFN-γ are present in higher concentrations in bovine colostrum than in mature milk; for example, IL-1Ra can be 180 times higher in colostrum than in mature milk [204], suggesting their importance as immunomodulatory factors in the newborn calf. Concentrations of these cytokines in colostrum can range from 77 to 5206 ng/mL [204]. There is still a lack of knowledge about the variation of other cytokines between the first milking and subsequent milkings.

Cytokines are essential in the immune response, but some cytokines need tight regulation; otherwise, the inflammatory process can have nefarious effects on the organism. For example, IL-6 plays important roles in inflammatory processes at the intestinal level but can also cause tissue damage, compromise the integrity of the intestinal barrier, and lead to systemic infections when overproduced [205,206]. IL-6 is mediated by the nuclear factor kB (NF-kB), which is involved in the pathogenesis of inflammatory diseases [207]. Bovine colostrum was able to reduce NF-kB activation and, consequently, IL-6 production in an in vitro model [206]. In another in vitro model, colostrum inhibited the NF-kB pathway in human colon cancer HT29 cells, protecting against intestinal epithelial cell inflammation [208]. These results are in agreement with Blais et al. [209], who observed a negative effect of bovine colostrum on the transcriptional activation of NF-kB. BC seems to have a strong capacity to increase the production of some cytokines and decrease others. It also appears to inhibit the production of cytokines that are present in higher concentrations in colostrum than in milk, such as IL-6 and INF-γ, probably as a means of regulating and establishing a balance between exogenous and endogenous cytokine levels. 

#### 4.1.6. Complement System

Colostrum and milk contain various proteins of the complement system [210,211,212]. They are involved in innate and specific immunity and play an important role in defence against pathogens. Complement proteins can be activated by the classical pathway, the alternative pathway, and the lectin pathway [210]. 

It seems that proteins related to the complement system are upregulated in colostrum compared to milk, such as clusterin (clearance of cellular debris and apoptosis in MFGM), complement factor B (related to the alternative pathway of the complement system), C3, C7, and C9 (classical pathway of the complement system) [211,212]. 

The newborn calf can acquire these proteins from colostrum but complement activity can also be found in the fetal serum right after birth. For example, C3 was not found in fetal serum and was only detected in calves between 1–3 days old [213]. In contrast, complement activity was not found in lambs at birth, only becoming detectable from day 1 (after colostrum consumption) and increasing until day 20 (end of the study) [214]. In this study, there were no differences in the complement activity, IgG, IgM, and chitotriosidase activity between lambs fed colostrum at 2 or 14 h post-natal. It has also been shown that the type of milk (goat milk or formula) after the colostrum feeding period can influence the activity of the complement system in goat kids [215]. It is also important to mention that temperature affects the expression of complement proteins (positively and negatively, depending on the proteins) [98,213]. For example, C1, C2, and C8 showed good heat resistance (56 °C), C7 showed moderate resistance, and C3 and C6 increased after heat treatment [213].

Classical and lectin pathways are compromised in the newborn calf, but complement-mediated cytotoxic functions normalize between days 7 and 28 after birth [16]. During this period, the incidence of neonatal enteric diseases is increased, so adequate transfer of immune factors through colostrum is essential to protect the calf while its immune system becomes competent. The combination of maternal and innate complement systems helps in the defence against microorganisms during the first days of life, but it is important to note that the effectiveness of the complement system in the GIT of the newborn calf is not clear due to the activity of proteases [210]. 

In this section, only those proteins in colostrum with the most scientific evidence that have important biological functions in the newborn calf have been mentioned, but there are many more that have not yet been extensively studied [212]. Colostrum has at least 253 proteins in the whey fraction, of which 36 are uniquely present in colostrum compared to milk [34,98,211]. Proteomic analysis of colostrum from different species could provide more knowledge about the factors of variation as well as the role of these proteins in the newborn [34,98,211]. 

### 4.2. Amino Acids

Amino acids (AA) can be divided into essential amino acids (EAA), non-essential amino acids (NEAA), free amino acids (FAA), and insoluble–proteome amino acids (IPAA; e.g., AA in casein) [216]. Lysine, methionine, phenylalanine, threonine, tryptophan, valine, leucine, isoleucine, histidine, and arginine are EAA for cattle. Calves receive AA via placental transfer for protein synthesis and energy utilization [15]. After birth, a continuous supply of AA is important for gluconeogenesis and for the high rate of protein turnover that is characteristic of the perinatal period [217]. Protein synthesis and nitrogen turnover are higher in the post-natal period (enteral feeding) than in the pre-natal period (parenteral feeding), which may indicate that the GIT is involved in protein metabolism [218]. Colostrum and transition milk are a source of EAA and NEAA. Calves fed colostrum and transition milk for 3 days had higher plasma EAA than calves fed colostrum and milk replacer and calves fed milk replacer alone [218].

A continuous supply of AA from milk is important, as AA intervene in regulatory and immunological activities that are essential for the growth and development of the young animal [216]. As mentioned above, several factors such as breed, age, and diet influence the protein concentration in colostrum and milk and thus the AA content. It has been shown that BC contains 0.32 g/L FAA and 4.2 g/L hydrolytic AA [219]. Total AA content decreases gradually from colostrum to mature milk, and FAA is present in lower concentrations than protein-bound AA [220]. 33 FAA and 29 IPAA in BC and 31 FAA and 30 IPAA were identified in milk [216]. In the FAA domain, 14 AA were significantly upregulated in colostrum compared to milk (histidine, threonine, isoleucine, leucine, methionine, phenylalanine, tryptophan, valine, glycine, proline, ethanolamine, hydroxyproline, 3-methylhistidine, and sarcosine) and six were significantly downregulated (glutamate, glutamine, alpha-aminoadipic acid, citrulline, beta-alanine, and 1-methylhistidine); in the IPAA domain, 6 AA were upregulated in colostrum (taurine, threonine, cysteine, arginine, serine, and alanine) and 6 AA were downregulated (lysine, sarcosine, ornithine, citrulline, alpha–aminoadipic acid, and beta–alanine) [216]. Taurine and valine were found in higher concentrations in colostrum (≈93–118 and 55 ug/mL as FAA; ≈18 and 379–398 ug/mL as protein-bound, respectively) and milk (12 and 3.8 ug/mL as FAA; 2.7 and 360 ug/mL as protein-bound, respectively) compared to other AA [216,219]. Isoleucine is also one of the most abundant FAA in colostrum (18.31 ug/mL) but not in milk (0.17 ug/mL) [216]. NEAA such as phosphorylethanolamine, ethanolamine, alanine, and glutamate also appear to be upregulated in colostrum and milk compared to other AA [216]. Some AA may only be present in colostrum or milk as FAA or bound to proteins. For example, free cysteine was detected only in colostrum and transition milk in the study by Zanker et al. [220]; however, in the study by Li et al. [216], it was not detected either in colostrum or in milk. In both studies, cysteine was present as a protein-bound AA in all samples. Glutamine was not found in colostrum but only in milk, and protein-bound glutamate was very high in colostrum in the study by Zanker et al. [220]. Li et al. [216] also found high concentrations of glutamate (mainly protein-bound) and lower concentrations of glutamine, but the authors considered colostrum from 0–7 days after birth, which may explain the differences between the studies. Conversely, Liang et al. [219] found much higher concentrations of glutamine than glutamate in colostrum. These differences could possibly be explained by breed and dietary factors, differences in proteolytic activity and microbial load of the samples collected in different studies, as well as what is considered colostrum by different authors. 

Valine, isoleucine, and leucine are branched-chain AA that are essential for the regulation and activation of signaling pathways, such as the mammalian target of rapamycin (mTOR) signaling pathway, which controls protein synthesis and degradation, hence resulting in muscle tissue development [221]. Thus, increased levels of these AA in colostrum may be associated with the upregulation of the mTOR system and the high neonatal protein turnover [222]. Taurine can contribute to innate immunity in pre-weaned calves by reducing pro-inflammatory signals in PMNL and by increasing their resistance to oxidative stress; however, supplementation above 8 ug/mL may lead to toxicity [223]. Hammon and Blum [218] observed an increase in plasma glutamate and a decrease in plasma glutamine in calves after colostrum and transition milk ingestion, demonstrating the important role of glutamine as a fuel for post-natal intestinal development. Glutamine plays an important role in the metabolism of macronutrients, serves as a precursor for molecular components, and intervenes in cell regulatory processes and immune functions [220]. Glutamine is a conditionally essential AA, and supplementation does not appear to be necessary except in specific catabolic situations [224]. Supplementation of pre-weaned calves with L-glutamine has been shown to reduce gastrointestinal permeability and biomarkers of physiological stress [225]. L-glutamine supplementation during weaning was also shown to have beneficial effects on growth performance, gut morphology, and physiological stress [226]. 

The AA content of colostrum appears to be necessary for various biological processes, particularly those related to protein turnover. However, even if EAA are not provided after birth, the calf can mobilize them from tissue stores until colostrum is provided for at least up to 24–25 h of life, after which plasma EAA levels normalize, unlike what happens with IgG, for example [220]. 

### 4.3. Fatty Acids

Although the general trend is similar, the lipid profile differs between colostrum, transition milk, and milk [227] (Table 4), highlighting possible specific needs of newborn calves [27]. Colostrum, like milk, is particularly rich in saturated fatty acids (SFA) and monounsaturated fatty acids (MUFA), with a lower proportion of polyunsaturated fatty acids (PUFA) [27,113]. Around 65.6–74.1% of the total FA are SFA, 24.5–28.4% are MUFA, and 3.88–4.28% are PUFA [27,227,228]. Compared to milk, colostrum seems to have a lower content of SFA, branched chain FA, MUFA, and conjugated linoleic acid (CLA) but a higher content of PUFA, ω-3, ω-6, and cholesterol (see Supplementary Table S1), and this is more pronounced in the first hours after birth [27,227,228,229]. In transition milk, the lipid profile generally gradually changes to a profile more similar to that found in milk. However, some lipids, such as high carbon number TAG, can maintain their initial concentration beyond the first milking [228]. Nevertheless, there is some variation within each group of FA, phospholipids, and TAG, so a general statement may not be entirely appropriate. There is disagreement in the literature regarding the lipid profile between colostrum and milk, so more research is needed to understand what factors may be involved in the differences observed among studies, such as supplementation during pregnancy, which appears to have a considerable effect [230,231]. There is a general agreement that palmitic and oleic acid are the most abundant FA in colostrum, but myristic and stearic acid are also present in higher proportions than the rest of the FA, although stearic acid is present in lower proportions in colostrum compared to milk [27,113,227]. This is also true for other species, with the exception of myristic acid [232]. The FA or FA groups present in higher concentrations in colostrum than in transition milk or milk are shown in Table 4.

Linoleic acid (LA) and alpha–linolenic acid (ALA) are essential fatty acids (EFA) [233], classified as n-3 and n-6 FA, respectively They cannot be synthesized by mammals and must therefore be ingested, and they are precursors to n-3 and n-6 PUFA, which are essential for metabolic regulation, cell membrane function, and gene regulation [231]. It is not clear whether LA and ALA are more concentrated in colostrum than in milk, but it appears that n-3 and n-6 fatty acids are more concentrated in colostrum (Table 5). However, further research is needed to clarify this due to the variability found between studies. Factors that can influence this variability in colostrum are not yet understood.

It appears that the placenta has different permeability to different FA; for example, supplementation of cows with docosahexaenoic acid or ALA during late gestation affected plasma levels of docosahexaenoic acid but not ALA in calves [234]. EFA and CLA supplementation altered the FA composition of skeletal muscle and adipose tissue in calves [235] and influenced their metabolism [236], growth, and feed efficiency [237] via placental, colostrum, or dietary transfer. 

Garcia et al. [238] found that pre-weaned calves supplemented with LA had lower plasma concentrations of acid-soluble protein and platelets, higher plasma n-3 FA, glucose, and IGF I, haematocrit and blood lymphocyte concentrations, increased IFN-γ production by peripheral blood mononuclear cells, and higher feed efficiency. Calves from cows supplemented with stearic acid (SFA) before parturition had higher dry matter intake and average daily gain [238] and higher plasma IgG and AEA [230]. Supplementation of cows with PUFA (LA or eicosapentaenoic and docosahexaenoic acid) resulted in higher colostrum IgG concentrations [239], and calves fed colostrum from supplemented cows also had higher plasma IgG levels and higher AEA [240]. The mechanisms underlying this increased IgG uptake are not fully understood. Nevertheless, the authors postulate that it could be related to an effect of the FA in the membrane of enterocytes [230], probably at the microvillar membrane, a specialized part in the luminal surface of the membrane, which is a phospholipid bilayer aggregated with specific proteins. However, it is important to note that immature cells found in newborns have different functionalities [241]. On the other hand, Hiltz and Laarman [242] found that calves fed colostrum replacer supplemented with 2.5% (*w*/*v*) butyrate decreased serum IgG concentrations and AEA, which may be related to the ability of butyrate to promote enterocyte differentiation and proliferation, leading to faster maturation and consequent loss of intestinal macromolecules absorption capacity, ultimately reducing IgG uptake [242,243]. 

A certain degree of oxidative stress is normal to occur during the fetal-to-neonatal transition; however, unresolved inflammation and insufficient antioxidant defence mechanisms can lead to functional and structural damage to cell components [244,245]. While generally, n-6 FA have pro-inflammatory proprieties, n-3 FA have anti-inflammatory capacity by either inhibiting the formation of eicosanoids, such as PGE2, PGF2α, and LTB4, or by forming anti-inflammatory lipid mediators, such as resolvins and protectins [246,247]. n-3 eicosapentaenoic acid can also be metabolized through cyclooxygenase and lipoxygenase pathways into prostaglandins, thromboxanes, and leukotrienes with lesser pro-inflammatory capacity [246]. Additionally, they can compete with AA for the cyclooxygenase and lipoxygenase enzymes, which are also necessary for highly pro-inflammatory prostaglandin formation (e.g., PGE2, PGF2, and PGD2), derived from the metabolization of n-6 arachidonic acid [246]. These mechanisms can reduce the transcriptional activation of NF-kB and the production of pro-inflammatory cytokines and enzymes [246]. Calves receiving colostrum supplemented with n-3 FA had increased plasma concentrations of n-3 FA and decreased oxidative stress but no change in health and growth parameters [245,248]. The authors suggest that continuous supplementation rather than one-time supplementation may have altered the results. However, Masmeijer et al. [249] found no benefit of continuous supplementation with n-3 FA on the health and growth of pre-weaned calves; furthermore, neutrophiles’ ROS production was increased in calves supplemented with microalgae compared to the control group. Further studies are needed to clarify the role of n-3 and n-6 FA in the neonate. The growth and immunomodulatory effects in calves vary depending on the type of n-3 FA, the dose, and the diet in which they are supplied, and may even worsen the outcome [249,250]. Overall, it seems to be beneficial to supplement the dam or calf with FA, but inclusion limits and the lipid source should be considered. However, it is clear that lipids are crucial for the newborn, highlighting the importance of the fat content of colostrum, which is sometimes overlooked in favor of the protein fraction.

### 4.4. Oligosaccharides 

Oligosaccharides (OS) are carbohydrates containing three to ten monosaccharides linked by glycosidic bonds and can be divided into two classes: neutral and acidic. While the neutral OS do not contain charged carbohydrate residues, acidic OS contain one or more negatively charged residues of sialic acid (the most prominent in BC is 5-N-acetylneuraminic) [251]. There have been 52 OS identified in BC and milk [252,253,254], but recently, another OS has been found in BC [255]. Sialylated oligosaccharides are the major OS in colostrum and milk, representing more than 70% and 50% of the total fraction, respectively [253]. 3′-Sialyllactose (3′SL) and 6′-Sialyllactose (6′SL), 6′-Sialyllactosamine (6′SLN), and Disialyllactose (DSL) are the most abundant in colostrum (Supplementary Figure S4) and are also more concentrated in colostrum than in mature milk, especially 3′SL [256,257,258] (Table 6). 

There are few studies quantifying OS in bovine colostrum and milk, mainly due to the difficulty of the analytical process [253]. Nevertheless, concentrations of the most abundant OS have been reported [24,256,257,258,259,260]. OS in bovine milk or colostrum can vary with breed [254,260], parity [24,258], days in lactation [253,258], hours postpartum [256], and heat treatment [259]. As seen in Table 6, 3′SL is the most abundant OS in colostrum, with considerable variation among studies. This may be related to the time of sampling after birth and individual genetic variation [252,258]. 3′SL was highly affected by heat treatment in comparison to other OS [259] and represents a major source of variation (see Supplementary Table S2 and Supplementary Figure S4).

Recent research has shown that OS in bovine milk have beneficial effects on neonates in several species. Milk OS may promote gut health by acting as a prebiotic for beneficial bacteria; increasing beneficial bacterial colonization of the surface of epithelial tissues; help defend against infection by acting as a decoy for pathogens, thus inhibiting pathogen adhesion to host target cells; by being able to modify epithelial glycan receptor expression; and also by competitive binding with the host cell surface receptor [261].

In an in vitro model, OS were shown to restore intestinal barrier function by promoting the formation of a mucus layer that reduces bacterial adherence, thereby increasing epithelial cell protection, and by reducing damage to the intercellular junction of intestinal epithelial cells [262]. OS ameliorated microbiota dysbiosis and intestinal barrier function in obese mice by increasing the abundance of *Lactobacillus* and reducing intestinal inflammation, as shown by the decreased expression of colonic TNF-α [263]. They can promote the growth of beneficial bacteria, which compete with pathogenic bacteria and produce metabolites such as bacteriocins and disrupt the acid–base balance in the gut, inhibiting the growth of pathogens [264]. It has also been shown that OS can help establish early colonization of beneficial bacteria, such as *Bifidobacterium*, in the newborn calf gut [259]. Bovine OS may also have an impact at the level of neurological tissues, as it has been shown to improve spatial cognition in premature pigs, with hippocampal upregulation of genes related to sialic acid metabolism, myelination, and ganglioside biosynthesis [265].

Compared to bovine, human colostrum has less protein and fat but more lactose [266]. This may be due to the different brain development between the two species, resulting in human offspring requiring more glucose available for neurological tissue development and functionality. Interestingly, the OS content of colostrum is similar in both species, but mature human milk can contain almost twice as much OS as mature bovine milk [267]. This similarity could be interesting for infant formulas. Although mammals are able to synthesize sialic acids for incorporation into neurological tissues, the rapid development of the infant may exceed the synthesis capacity; hence, the higher concentration in human milk [268]. Elephant milk has similar concentrations of lactose to bovine milk, which are lower than human milk, but the content of OS is higher than in bovine milk and three times higher than in human milk, with a very different profile [269]. OS in primate milk is more complex and diverse than in non-primate animals [268]. To date, only chimpanzees, bonobos, and Asian elephants have specific combinations of OS characteristics of the human species [270]. This information highlights the importance of OS in more neurologically advanced species, as well as their importance for neurodevelopment and cognition in neonates [267], but more research is needed to understand the functions of OS in neonates and how different OS interact in different species.

### 4.5. Endocrine Factors

#### 4.5.1. Hormones

Although there has been some considerable research into the hormones present in milk in the decades of the 70s and 90s [271], research into the hormone concentrations in colostrum is limited. Colostrum appears to be more concentrated than milk in androstenedione, estrone, oestradiol, cortisol, cortisone, GnrH, GH, prolactin, TRH, insulin, glucagon, leptin, adiponectin, and motilin than milk, and the opposite seems to be true for parathyroid hormone-related peptide (PTHrP), testosterone, progesterone, and rT_3_ (Table 7). There are other hormones for which this difference between colostrum and milk is not yet clear, such as corticosterone, GHIH, oxytocin, bombesine-like peptide, gastrin-releasing peptide, neurotensin, vasoactive intestinal peptide, calcitonin, melatonin, and erythropoietin. Some hormone concentrations in milk are influenced by lactation phase, pregnancy status, season, diurnal patterns, physiological state of the animal, and also differences between analysis methods [272,273,274,275,276], making direct comparisons with colostrum difficult. 

The exact role of these hormones in the neonatal ruminant is not fully understood, but it is known that they can be absorbed into the circulation and that they may contribute to the maturation of the gastrointestinal, endocrine, and immune systems [277]. This is particularly relevant due to the immaturity of the newborn GIT, which allows the passage of these hormones into the circulation and, thus, results in a systemic effect [84]. They can be found in higher concentrations in colostrum or milk than in maternal blood plasma, highlighting their importance for the offspring [271,276]. However, although leptin concentrations are higher in colostrum than in milk (Table 7), there is no evidence of leptin absorption by the neonatal calf, as occurs in other species such as rats and humans, where this hormone has systemic effects related to food intake, insulin-dependent glucose metabolism, intestinal maturation, and thermogenesis [30].

Interestingly, some steroid hormones are present in higher concentrations in human colostrum than in BC, which could be related to the evolution of the mammalian brain, as they regulate cellular mechanisms such as synapse formation, dendritic arborization, and cell turnover, and generally contribute to physiological, behavioral, and cognitive functions [278,279]. However, steroid concentrations in foods of animal origin can be of concern, making it important to assess hormone concentrations in BC, as it is one of the foods with higher levels of these hormones [280]. 

These peptides exert many different essential functions in the newborn, such as regulating tissue growth and differentiation (promoting: GH, GH-releasing factor (GRF), IGFs, EGF, TGFα; inhibiting: somatostatin (growth hormone-inhibiting hormone—GHIH), and TGF-β), metabolism and thermogenesis (TRH, TSH, T_3_, and T_4_), energy status and glucose metabolism (insulin, glucagon, adiponectin, glucocorticoids, catecholamines), and blood serum calcium levels (parathormone (PTH) and parathyroid hormone-related protein (PHrP)) [15,277,281,282,283].

Neohormones, like oxytocin and relaxin, are present in milk and are involved in the regulation of vital mammalian traits, such as internal fertilization, pregnancy, and lactation [284]. Milk-derived relaxin (in colostrum) has been shown to influence the development of the neonatal reproductive system in female pigs [285]. The transfer of these bioactive factors, which act as signaling molecules in neonatal tissues, through nursing is known as lactocrine signaling [285,286]. Although relaxin is present in the milk of different species, it appears that the gene encoding ovarian relaxin-2 is deleted in bovine and ovine species [287]. Calcitonin is a hormone produced by the thyroid gland that lowers blood calcium by inhibiting the reabsorption of bone calcium and increasing urinary calcium loss [288]. An inhibitory effect of prolactin release by calcitonin has also been reported [277]. Although calcitonin has been mentioned in many publications as present in bovine colostrum [38,84,165,184,289] and milk [271], the authors could not find any research showing its concentrations in cow colostrum or milk, as opposed to human and rat milk [276,277,290].

**Table 7:** Reported hormone concentrations in bovine colostrum and milk.

Hormone	Colostrum	Milk	References
Gonadal Hormones			
Androstenedione (ng/mL)	0.18–8.36	0.1–3.5	[274,279,280,291]
Estrone (E1) (ρg/mL)	1300–31,070	0.6–159	[273,274,279,280,292]
17α-Estradiol (ng/mL)	8.6	0.03	[274,280]
17β-Estradiol (E2) (ρg/mL)	300–7010	0.3–14.0	[273,274,279,280,292]
Estriol (E3) (ρg /mL)	<3000	9.0–31.0	[280,292]
Testosterone (ng/mL)	0.1–≈1.6	20–120	[279,280,291]
Progesterone (ng/mL)	2.62–6.46	2.13–15.49	[274,279,280]
Adrenal gland hormones			
Corticosterone (ng/mL)	?	2.92 ± 0.26	[293]
Cortisol (ng/mL)	1.71–4.4	0.35–1.28	[279,293,294]
Cortisone (ng/mL)	2.16 ± 1.71	0.11–0.51	[274,279]
Hypothalamus-Hypophyseal Hormones			
Gonadotropin-releasing hormone (GnRH) ^a^ (ng/mL)	11.78 ± 0.72	0.5–3.0	[295]
Growth hormone (GH) ^b^ (ng/mL)	0.17–1.4	<0.03–<1.0	[11,296,297,298]
Growth hormone-inhibiting hormone (GHIH) ^c^ (ρM)	?	19.0 ± 6.0	[299]
Oxytocin (ρg/mL)	?	8.0–10.0	[300]
Prolactin (ng/mL)	280–800	3.7–57.0	[11,301,302,303]
Thyrotropin-releasing hormone (TRH) (ng/mL)	0.16 ± 0.03	0.05	[295]
Brain-Gut Hormones			
Bombesin-like (related to gastrin releasing peptide) (ng/mL)	?	1.17 ± 0.89	[304]
Gastrin releasing peptide (nM)	?	1.4 ± 1.0	[299]
Neurotensin	?	?	
Vasoactive intestinal peptide (ρM)	?	16 ± 9.0	[299]
Pancreatic hormones			
Insulin (ng/mL)	35.4–65	1.0	[11,305]
Glucagon (ng/mL)	0.16	0.01	[11]
Thyroid Gland Hormones			
Calcitonin	?	?	
Triiodothyronine (T_3_) (ng/mL)	<0.31–2.02	0.21–0.41	[296,306,307,308]
Reverse Triiodothyronine (rT_3_) (ng/mL)	0.57 ± 0.06	3.48–91.1	[307,308]
Thyroxin T_4_ (ng/mL)	0.12–1.9	0–0.67	[296,306,307,308,309]
Other hormones			
Parathyroid hormone-related protein (PTHrP) (ng/mL)	26.0–56.0	59.0–168.0	[310,311]
Glucagon (ng/mL)	≤0.16	0.01	[11,297]
Relaxin	-	-	
Melatonin (ρg/mL)	?	4.71–41.94	[275,312]
Erythropoietin	?	?	
Leptin (ng/mL)	13.9–30	4.4–6.1	[272,313]
Adiponectin (µg/mL)	56.1 to 75.9	0.61	[314,315]
Motilin (ng/mL)	0.23 ± 0.06	0.03 ± 0.02	[316]

Values refer to mean values reported in research papers. When a single value was found, mean and standard deviation was reported instead. ^a^ Also referred to as Luteinizing hormone-releasing hormone (LH-RH). ^b^ Also referred to as Somatotropin. ^c^ Also referred to as Somatostatin.

#### 4.5.2. Growth Factors

After immunoglobulins, growth factors are probably the most studied bioactive components in BC [2,73,124,298,317,318,319,320]. There are about 50 different polypeptides that can modulate the growth, maturation, and function of the GIT [11,44,321]. Insulin-like growth factor (IGF) 1 (IGF-I) and 2 (IGF-II), transforming growth factor-β (TGF-β), epidermal growth factor (EGF), β-cellulin (BTC), fibroblast growth factor 1 and 2 (FGF1 and FGF2), platelet-derived growth factor (PDGF), and vascular endothelial growth factor (VEGF) are probably the most important growth factors in colostrum [7,321,322,323]. See Baumrucker and Macrina [303] for cow colostrum and milk concentrations. Growth factors have a certain degree of thermal tolerance, since they can withstand temperatures up to 60 °C for 30 min [298] or 60 min [324]. However, the need for caution when heat-treating colostrum is reinforced, as to the authors’ knowledge, there is no information beyond these ranges. These factors also need to survive digestion, retain biological activity, have receptors in the GIT, or be absorbed to influence the neonate. The GIT has site-specific receptors in different regions, so it is expected that bioactive components in colostrum will have different effects in different regions of the GIT [166]. The epithelial cells have a high turnover rate, and IGFs are key regulators of cell growth and differentiation [325].

Compared to human colostrum, BC is particularly high in IGFs and low in components of the EGF family [166]. EGF binds to the epidermal growth factor receptor (EGFR), which increases intracellular tyrosine kinase activity, activating signaling cascades that ultimately promote cell proliferation and angiogenesis and reduce apoptosis [326]. Other EGFR-related ligands are TGF-β and BTC [166]. TGF- β is associated with immunomodulatory activities, inflammatory responses, oncogenesis, and proliferation of intestinal cells [44,323,327]. IGF concentration is also higher in colostrum than in blood [166]. The IGF system is composed by IGFs and IGF-binding proteins (IGFBPs) [317]. IGFs are synthesized in the liver in response to the growth hormone (GH) [328], and some IGFBPs are synthesized in the lactocytes of the mammary gland [329]. IGFs and IGFBPs are highly concentrated in prepartum secretions and colostrum and decline rapidly in subsequent milkings [11]. While EGF may be more important in the human and porcine species, it can be suggested that IGFs are more important for the ruminant neonate, as they are present in higher concentrations in ruminant colostrum [317]. Whether the growth factors in colostrum and milk are exclusively directed to the offspring or to mammary gland development (e.g., cell proliferation and differentiation) is not entirely understood. Heifers have higher circulatory concentrations of IGFs than older cows, which vary throughout the pregnancy–lactation cycle [330]. They are higher in the dry period, decrease at the onset of lactation, and then gradually increases until late lactation [331]. They are present in much higher concentrations in colostrum than in milk (IGF-I around 3000 and 5–50 ug/L, respectively) [331]. IGF-I in colostrum is also mainly in a free form, which leads to higher bioavailability of IGF-I [331]. So, it seems that these growth factors in colostrum are mainly directed to the neonate but not exclusively. Nevertheless, the neonate calf is also capable of synthesizing IGFs in different tissues [166]. The components of the IGF system have different regulatory properties in GIT epithelial cells. IGF-I appears to be more involved in mitogenesis, which promotes enterocyte proliferation and regeneration, and IGF-II may be more related to enterocyte differentiation promoting functions [325]. 

Delaying colostrum feeding can negatively affect some metabolic and endocrine traits of the newborn. Calves fed colostrum on day 1 had increased circulating glucose, albumin, insulin, and IGF-I concentrations compared with calves fed on day 2 of life [332]. Fischer-Tlustos et al. [333] showed that calves fed colostrum right after birth (0 h) had increased GIT development in terms of some histomorphological parameters like villi height, crypt depth, and surface area index, compared to calves fed 12 h after birth at 51 h of age. Smaller differences were observed when compared to calves fed 6 h after birth. However, there were no differences in the serum IGF-I concentrations of calves with respect to feeding time but it was 29% higher at 48 h than at 0 h after birth (312.8 ± 14.85 vs. 241.9 ± 14.06 ng/mL) [333]. On the other hand, extending colostrum feeding can promote GIT maturation, but similar results can also be obtained with a mixture of colostrum and whole milk (1:1) [334], which has a composition similar to transition milk. Even after gut closure and with theoretically increased proteolytic activity, calves fed colostrum or a mixture of colostrum and whole milk had a greater surface area of the GIT and increased villi height in the proximal and distal jejunum than calves fed whole milk, and ileal crypts tended to proliferate more with a mixture of colostrum and whole milk than with whole milk alone, but crypt depth did not differ [334]. However, calves fed colostrum had only minimally increased plasma GLP-2 and serum IGF-1 compared to calves fed whole milk, which the authors believe may be related to inadequate nutrient intake [334]. In an in vitro study, it was shown that amino acid deficiency reduced intestinal epithelial cell reconstitution through a decrease in TGF-β production [335]. The maternal diet during gestation can also affect the GIT development of the newborn, as lambs from ewes supplemented with folic acid during pregnancy were shown to have increased IGF-I expression, small intestine weight/live body weight ratios, and intestinal muscularis thickness [336]. In fact, growth factors present in colostrum may be able to influence the neonate’s growth performance until later in life. A study by Buranakarl et al. [337] showed that kids fed colostrum with higher concentrations of IGF-I (>518.3 ng/mL) had increased body weight gain at the end of the first month than kids fed colostrum with lower concentrations of IGF-I (≤518.3 ng/mL). Unfortunately, milk composition was not analyzed, since it could have affected the kids’ development. It is important to note that IGFs are not the only molecules intervening in intestinal maturation, as it is a combination of several bioactive components [317].

### 4.6. Nucleic Acids

#### 4.6.1. MicroRNA

A review on microRNA (miRNA) in bovine colostrum has recently been published [13], and this is a very new area of study within the colostrum field; therefore, we will only briefly describe these molecules in colostrum and milk and discuss some recent studies on their effect on the newborn calf. MiRNAs are a group of small (18–25 nucleotides in length), endogenously expressed, non-coding RNA molecules that act as regulators of gene expression and other cell-related regulatory functions, like survival, proliferation, apoptosis, tumor growth, and metastasis [338,339]. It has also been shown that miRNAs can act as regulators in many immunological pathways [12,340], in wound healing, and in infection processes [341,342]. Other non-coding RNA molecules include transfer RNAs (tRNAs) and ribosomal RNAs (rRNAs) [343].

It is suggested that miRNAs are produced in the mammary gland and transferred to milk and colostrum [163,344,345], protected within extracellular vesicles (EV), such as exosomes. Colostrum seems to have a higher concentration and expression of different miRNA molecules than transition milk and milk [163,346,347], and some novel miRNAs are still being discovered [344,348]. EVs are cell-secreted membrane-encased vesicles that contain proteins, miRNAs, mRNAs, and lipids, depending on their source, and are associated with various physiological and pathophysiological processes [342,349]. This allows miRNAs to be resistant to acidic conditions (pH 2 at 37 °C for 1 h) and to RNases [346], making them resistant to the GIT environment and allowing absorption in the small intestine [13]. This finding contributes to the theory that miRNAs in colostrum and milk act as signaling molecules between the mother and the offspring [13]. However, according to Hue et al. [345], the miRNAs present in the newborn calf’s bloodstream are not colostrum-derived but rather from an endogenous source. The study by Kirchner et al. [349] also did not confirm that the miRNAs present in the EVs of colostrum either entered the circulation of the newborn calf, remained in the intestinal epithelium, or were rapidly transported to other tissues and were therefore not present in the bloodstream at the time of analysis. These results do not indicate that miRNAs from colostrum are absorbed by the newborn calf.

Depending on the phase of lactation, different miRNAs may be expressed [13]. It seems that during colostrogenesis, there are predominantly expressed miRNAs associated with immune pathways, while during the rest of the lactation, the miRNA expressed are more related to milk synthesis [344,350]. Colostrum is also more concentrated in miRNAs than in transition and mature milk [347]. The same applies to breed differences; compared to beef heifers, the mammary glands of dairy heifers had a more pronounced downregulation of miRNAs involved in the inhibition of genes related to the maintenance and activity of mammary stem cells, presumably required for intensive regenerative processes during puberty and pregnancy [351]. While there are miRNAs that are commonly expressed in colostrum and milk of different species, like miR-30a-5p, miR-22-3p, and miR-26a, which are related to immune functions, there is also significant variation between species [352]. It was found that miRNA abundance was not affected by heat treatment at 60 °C for 60 min and that miRNAs were higher in frozen samples than in raw samples [347].

Some miRNAs have a higher expression level in BC than in milk, such as miR-142-5p, miR-150, miR-155, miR-181a, and miR-223 [163,345]. Ma et al. [353] found that colostrum with very different IgG levels (62.8 ± 3.6 and 256.5 ± 5.7 mg/mL) had a similar expression profile of miRNAs present in small EVs, which contributes to the statement that colostrum with a higher concentration of IgG does not necessarily indicate higher concentrations of the many other BC reviewed in the present article. Nevertheless, studies correlating IgG and other BC are still lacking. Only one miRNA was more abundant in high-IgG colostrum—miR-27a-3p—which is associated with osteogenic differentiation in pre-osteoblasts [353] and with glycaemic and lipid status in women with gestational diabetes mellitus [354].

The importance of miRNAs to newborn health is clear, and it is also clear that these molecules are highly expressed in colostrum; however, it is not clear if their action is dependent on uptake into the bloodstream or if they may act in the GIT. In any case, it seems that they can act as regulators of the mucosal immune system [355], which reinforces their importance as immune modulators of GIT. 

#### 4.6.2. Nucleotides and Nucleosides

Nucleotides are monomers that constitute the basic building blocks of nucleic acids (RNA and DNA). They consist of a nitrogenous base, a pentose sugar, and a phosphate group. Nucleosides are essentially nucleotides without the phosphate group. Nucleobases are the nitrogenous bases that are the basic units of the genetic code (adenine (A), cytosine (C), guanine (G), thymine (T), and uracil (U)). They are essential for cellular function, acting as mediators of chemical energy transfer, signal transduction, and growth regulators [356,357]. 

Nucleo(s/t)ides are present in milk and colostrum in the sub-milligram per liter range and belong to the non-protein nitrogen (NPN) fraction [358]. The concentration of these compounds shows little variability during lactation, except during the first few days [358,359]. Immediately after birth, the concentration of nucleotides in colostrum is low but rises to a maximum 24–48 h later and then gradually decreases [360,361]. Nucleosides are generally found at lower concentrations in milk than nucleotides. They are found in higher concentrations in colostrum without a clear maximum and generally decrease to a constant level by 3 weeks after parturition [359]. Uridine and uridine 5′-monophosphate (5′UMP) are the most abundant nucleo(s/t)ides in colostrum, with concentrations ranging from 50.6–102.4 and 13.33–143.7 µmol/dL, respectively [360,361]. Concentrations and variations during lactation are species-specific, e.g., uridine levels in ewe milk can be four times higher than in cow and goat milk at about 24 h postpartum [360]. Orotic acid is present in much higher concentrations in cow milk compared to ewe and goat milk and appears to increase during the first 2 months of lactation [360]. Gill et al. [357] found a difference in the total base concentration (nucleotides and nucleosides) in colostrum collected in summer and winter (62.1 ± 6.2 and 258.7 ± 6.8 µmol/dL, respectively), with a notable difference between the concentration of 5′UMP in summer (1.2 ± 0.0 µmol/dL) and winter (143.7 ± 8.5 µmol/dL). There is still very little information on the variation of nucleo(s/t)ides in ruminants’ colostrum, and there seems to be a significant impact of species, breed, season, and analytical methods on the differences obtained so far [357,359,360,361]. 

Nucleotides, especially those with an uracil nucleobase, contribute to the calf’s biological functions. Mashiko et al. [362] showed that supplementation with 2 g/d of 5′UMP had a positive impact on the immune status of calves aged 4–42 days. In Katoh et al.’s study [363], an effect of 2 g/d of 5′UMP on the endocrine status of calves 4–14 days old was observed. Oral administration of 5 g of a nucleotide supplement (5′CMP, 5′UMP, 5′AMP, and 5′GMP) improved growth performance, intestinal morphology, and oxidative status in pre-weaned calves [364]. Supplementation of sows with uridine and cytidine (Ur:Cy = 1:1) reduced birth mortality, increased piglet birth weight, and modulated gene and protein expression of enzymes involved in lipid metabolism in the liver of neonatal piglets [365]. Maternal supplementation with yeast-based nucleotides containing 5′UMP increased litter weaning weight and decreased diarrhea rate, promoted ileal villus development, increased secretory IgA in the ileum and jejunal, and increased jejunal and ileal expression of interleukin IL-17, IL-8, IL-1β, IL-10, and TNF-α in piglets [366]. However, in another study where sows were supplemented with a yeast-based nucleotide without 5′UMP, there was no effect on sow or piglet performance [367]. Including 10 and 20% of a nucleotide mixture (NuPro, Alltech Inc., Nicholasville, KY, USA) in the milk replacer formula of male calves worsened growth performance and did not improve health status [368]. Feeding calves a yeast-based nucleotide mixture (NuPro, Alltech Inc., Nicholasville, KY, USA) resulted in improved intestinal function and morphology, but feeding purified nucleotides (80 µmol/L of AMP, 64 µmol/L of CMP, and 374 µmol/L of UMP) did not and resulted in lower fecal beneficial bacteria, higher harmful bacteria, fecal water loss, and calf dehydration [369]. A reduction in *Lactobacillus* spp. was also observed in calves supplemented with nucleotides (yeast-derived; 500 g/t of milk replacer; Ascogen, Chemoforma, Switzerland) [370]. Most studies on nucleotide supplementation have tested pre-weaned calves, but it appears that post-weaning nucleotide supplementation may result in better growth performance [371,372]. It may be of interest to study the effect of nucleotide supplementation, especially uracil-based, in the very first days of life, given the good results of maternal supplementation and the natural variation of nucleo(s/t)ides in BC.

## 5. Cells and Microorganisms in Bovine Colostrum

### 5.1. Cells

The cell fraction of colostrum can be divided into epithelial cells (lactocytes), erythrocytes, and leukocytes [9,162]. The mammary gland’s epithelial cells contribute to host defence by expressing pathogen recognition receptors (e.g., toll-like receptors) and by synthesizing proteins with antimicrobial properties such as lactoferrin, β-defensin, and lipopolysaccharide-binding protein [9]. Epithelial cells are present in the bovine colostrum at 2 to 15% of the total cell count [373]. In contrast, epithelial cells make up more than 20% of the total cell count in sow colostrum [8]. The leukocyte fraction consists mainly of lymphocytes, macrophages, and neutrophils, and its concentration depends on diet, age, breed, and physiological and individual conditions [8,373,374]. For example, it has been shown that heifers have lower activity of colostral immune cells than cows with three or more lactations [375]. On the other hand, Kampen et al. [376] found no significant effect of cow health status on colostral lymphocyte concentration. In Park et al.’s study [377], colostrum collected 48 h before and after parturition differed in the percentage of leukocytes, with an increase in all subpopulations except macrophages, which increased, suggesting a decrease in leukocyte count with the onset of lactation, similar to other components in BC; however, only two cows were sampled in the 48 h prepartum period. In contrast, in pigs, the percentage of T lymphocytes, B lymphocytes, and macrophage/monocyte subsets in colostrum did not differ between 0 and 8 h postpartum [378].

Colostrum contains approximately 10^6^ leukocytes/mL, and macrophages and neutrophils are the major fractions of colostral cells, followed by lymphocytes (Table 8) [347,373,377,379]. In the lymphocyte fraction, T lymphocytes represent a higher proportion of the total cell count than B lymphocytes, at 16 and 10.7%, respectively [377,379,380]. Although these proportions appear consistent across studies, there is still considerable variation in the number of cells. The total cell count, usually referred to as the somatic cell count (SCC) of colostrum, is around 2.3 × 10^5^ and 5 × 10^5^ cells/mL [374,379] but can range from 1.2 × 10^5^ to 1.9 × 10^6^ cells/mL [107,381]. The specific influence of factors on these variations has yet to be entirely understood. 

Significant effects of freezing and heat treatment on colostral cell viability have been reported. The rapid freezing of colostrum in liquid nitrogen, followed by a slow thawing, resulted in lysis of the cells, which led to an inability to detect these cells under the microscope [385]. Rapid freezing of colostrum on steel plates pre-cooled to −80 °C followed by heat treatment at 50 °C also resulted in cell lysis [386]. Similarly, no percentage of viable cells was found in pooled colostrum frozen at −20 °C for a period between 24 h and 3 months and then thawed (37 °C) prior to administration, in contrast to fresh colostrum, which had 24 ± 8% viable cells [387]. Similar results were found in Chandler et al.’s study [347], with no viable cells after freezing at −20 °C overnight or heat treatment (60 °C for 60 min), but there were more than 80% viable cells in fresh colostrum cooled on ice and stored at 4 °C overnight. In contrast, frozen (−20 °C) porcine colostrum had reduced numbers of lymphocytes (CD79a+) and conventional B cells (SWC7+CD5-) but no macrophages, granulocytes, and NK cells [378]. Similarly, feeding fresh or frozen colostrum did not significantly affect the neutrophil and monocyte activation in newborn dairy calves [388]. Heat treatment (60 °C for 60 min) reduced the SCC of bovine colostrum by 36% [320]. Martínez et al. [389] studied the effect of different treatments on the SCC of ewe milk analyzed by the Fossomatic method and concluded that freezing milk at −20 °C only reduced the SCC when azidiol was used as a preservative and when the milk was heated to 60 °C, whereas unpreserved milk or milk preserved with bronopol or potassium dichromate and analyzed at 40 °C did not affect the SCC. Significant differences in SCC between fresh and frozen BC were also not found in another study [61]. It can be assumed that freezing may cause cell lysis, but they can still be counted in the Fossomatic method, as according to Chandler et al. [347], intact (but not viable) cells and cell components are still visible after freezing. Freezing and heating may, therefore, affect cell viability in colostrum.

Colostrum management practices, such as giving priority to fresh colostrum, which can be refrigerated for about 2 days (or even longer if a preservative such as potassium sorbate [4,22] is added), rather than freezing it immediately if more cows are likely to calve in the next few days, could help preserve maternal cell function in colostrum. Another possibility could be the fermentation of colostrum as a preservation method, but there is currently no information on the cell viability of fermented colostrum. 

Leukocytes are absorbed by the intestinal epithelium of the newborn, allowing them to migrate to Peyer’s patches and mesenteric lymph nodes or to enter the systemic circulation and eventually reach other organs associated with the immune system, such as the liver and spleen, normally within 24 h after colostrum ingestion [373,390,391]. To be absorbed, leukocytes must undergo phenotypic changes provided by the colostrum environment in the mammary gland [391].In the swine species, there is a maternal–neonatal recognition that does not allow the passage of colostral cells or peripheral blood leukocytes from other sows through the piglet’s intestinal wall [8], but it does occur in primates [392] and sheep [393] and probably in cows, although this is not clear as in most studies, calves were fed colostrum from their respective dams. It is important to identify this issue, as many farmers feed calves with colostrum from a colostrum bank rather than from the dam. Nonetheless, in one study where calves were fed pooled colostrum or colostrum from another dam, passive immune transfer, morbidity, and weaning weight did not differ from calves fed maternal colostrum [394].

At birth, the newborn calf has a naive innate immune system; a deficient and immunosuppressive state is evidenced by increased expression of TGF-β1 and TGF-β2, together with reduced functions of phagocytosis and platelet aggregation [16]. Thus, immunocompetent colostral cells (as well as immunoglobulins and other immune factors present in colostrum) are necessary to induce cell-mediated responses and enhance both innate and adaptive responses. 

It is likely that maternal leukocytes migrate to different tissues depending on their ability to express the CD62L receptor. Leukocytes with little or no expression of CD26L are likely to migrate to peripheral non-lymphoid tissues where they act as memory cells; leukocytes expressing higher levels of CD26L expression are more likely to migrate to secondary lymphoid tissues where they can act as regulators of the newborn’s specific immune responses [391]. In fact, memory-activated T-cell phenotypes are the most common in colostrum [8,391]. 

Maternal colostral cells allow for more rapid development of adaptive immunity by enhancing the antigen-presenting capacity of monocytes and lymphocytes, as indicated by increased expression of MHC class I molecules [395,396]. Maternal colostral cells also have a long-term effect on the development of the calf’s immune system, improving responses to vaccines [397]. Maternal vaccination may improve neonatal defences against certain pathogens, as transfer of antigen-specific lymphocytes through colostrum can occur with antigens to which the dam has been exposed [373,386], but there are still few studies in bovine species on maternal–neonatal transfer of antigen-specific immunity. 

Colostrum affects the B-cell lineage of the newborn calf [398]. Exposure to maternal commensal microbes in utero or spontaneous production of low-affinity natural antibodies causes a calf that has not received colostrum to have IgG-positive cells in the lymphoid tissues, whereas calves fed colostrum do not [398]. This occurs because the colostral immune system can suppress or eliminate IgG-positive B cells but does not affect IgM- and IgA-positive cells in the mesenteric lymph nodes, which may play an important role in mucosal immunity in the early life of the calf [398]. BC also has a much higher concentration of IgG than IgM and IgA, which may also contribute to this suppression of neonatal IgG production but not IgM and IgA. This phenomenon may be necessary for transferring maternal components (which would otherwise be foreign to the neonate), which also results in prolonged tolerance to maternal immunoglobulins, increasing their longevity during the development of the innate and acquired immune systems [398].

### 5.2. Microorganisms 

Feeding colostrum increases gut bacterial colonization in the newborn calf [399]. Bacteria can reach the cow’s mammary gland exogenously, specifically from the maternal skin, or endogenously with an immune-related translocation of intestinal bacteria to the mammary gland, referred to as the entero–mammary pathway [400]. The former can explain the presence of aerobic microorganisms, and the latter can explain the presence of anaerobic microorganisms in colostrum or milk. It is hypothesized that the aim of this process is to ‘train’ or ‘educate’ the newborn’s immune system to recognize commensal microorganisms and develop an appropriate response [400]. 

The microbial quality of colostrum is usually assessed using total plate count (TPC) and total coliform count (TCC), which provide an overall assessment of hygiene conditions from milking to feeding. It is difficult to establish a mean value for the TPC of BC because there is a large variation between published studies. Mean TPC in raw colostrum can range from 250,000 [401] to 1062 ufc/mL [402] ufc/mL,. Mean TCC can range from 12 ufc/mL [61] to 63,000 ufc/mL [387]. Elevated TPC and TCC are associated with colostrum contamination. Current targets are a TPC below 100,000 cfu/mL and a TCC below 10,000 cfu/mL [403]. While coliforms are essentially fecal bacteria that can include *E. coli*, TPC includes a wide variety of bacteria, including beneficial bacteria. Therefore, a higher TPC does not necessarily indicate poorer microbial quality. Other recommendations from the same reference include streptococci and staphylococci below 50,000 cfu/mL [403]. 

Heat treatment of colostrum is aimed at reducing pathogenic bacteria. It was found that heating colostrum at 60 °C for 60 or 120 min reduced TPC and pathogenic bacteria without affecting IgG concentration [7,404]. Other combinations of time and temperature above 60 °C resulted in a significant loss of IgG concentrations [7,404]. Heat treatment can effectively reduce TPC and TCC, but staphylococci appear to be more resistant to heat treatment [320].

Colostrum also contains beneficial bacteria, like lactic acid bacteria (LAB), but current recommendations are based only on pathogenic bacteria. While heat treatment significantly reduces TPC and TCC [320,401], there is still a gap in the knowledge of the interaction between beneficial and pathogenic bacteria in raw, frozen, and heat-treated colostrum. Using quantitative real-time PCR analysis, detectable bacteria were only present in untreated colostrum samples, from which *E. coli* represented 70.6% of the total bacteria, whereas no bacteria were detected in heat-treated (60 °C/60 min) samples, but it was shown that calves fed heat-treated colostrum 6 h after birth had higher intestinal colonisation with *Bifidobacterium* [399]. In another study, a significant reduction in LAB (lactococci and lactobacilli) counts was found when heat treatment (treatment 1–56 °C/60 min or treatment 2–63 °C/30 min) was applied to raw caprine colostrum [405]. Beneficial bacteria can compete with pathogens and improve the structural integrity of intestinal epithelial cells by increasing transepithelial resistance, promoting tight junction stability and mucin expression [406]. LAB isolated from colostrum showed good in vitro antimicrobial activities and antioxidant power [407]. LAB supplementation improved body weight gain, feed conversion, and fecal condition in calves [408,409]. While pathogenic bacteria can reduce IgG absorption in the neonatal gut by binding to bacteria or by competing for epithelial receptors, the role of beneficial bacteria in this process is not well understood [410]. 

Culture-independent microbiome studies have shown that the BC microbiota is dominated by four phyla: Firmicutes, Bacteroidetes, Proteobacteria, and Actinobacteria [14,410,411,412]. The top 10 genera in BC are listed in Table 9. Of these 10 genera, *Acinetobacter*, *Pseudomonas*, and *Staphylococcus* are the most frequent in BC. Factors such as season, breed [410], parity [14], antibiotic administration [413], and mastitis [14] can affect the colostrum microbiota. At the phylum level, colostrum core microbiome is relatively stable, at the genus level, there is some variation that is not fully understood, and at the species level, there is considerable variation [411].

The genus *Acinetobacter* comprises more than 50 Gram-negative coccobacilli species, from which the majority are non-pathogenic environmental organisms [415]. *A. baumannii* is the best known species of this genus and has clinical significance in both human and veterinary medicine, as it can be associated with different types of infections in different species and due to its ability to accumulate antimicrobial resistance [416]. Patangia et al. [413] have confirmed that *Acinetobacter* had a high abundance in the colostrum of the cows that received antibiotics as part of the dry therapy. They are common in the environment and are therefore likely to be present in the cow’s GIT, allowing them to migrate into colostrum, but it is not yet understood whether they have a specific role in the mammary gland or in the GIT of the neonate, as they are commonly abundant in different studies.

*Pseudomonas* and *Staphylococcus* can also be pathogenic and appear to be positively correlated in colostrum, as well as *Chryseobacterium* [411]. In contrast, they were negatively correlated with the *Bacteroidales-S24-7-group*, which are beneficial bacteria [411]. There also appears to be a relationship between the concentration of IgG and the prevalence of certain microorganisms in colostrum. For example, the genera *Lactococcus* and *Carnobacterium* (beneficial bacteria) were more abundant in colostrum with an IgG concentration above 100 g/L than in colostrum with an IgG concentration below 50 g/L [410]. In contrast, a species of the *Enterobacteriaceae* family (coliforms) was 97 more abundant in colostrum, with an IgG concentration of less than 50 g/L compared to colostrum with a higher IgG concentration (>50 g/L) [410]. It is not yet understood whether IgG is lower or higher depending on the microbiome or the opposite, but it seems plausible that a combination of both may occur. Lima et al. [14] found that bacteria with a pathogenic tendency were more abundant in the colostrum of multiparous cows compared to primiparous cows and that primiparous cows had a richer microbiota, but primiparous cows with lower diversity might be more susceptible to future disease events. These findings highlight the impact that the microbiome in colostrum and milk may have on mammary gland defence and consequently on the calf’s mucosal defence. Calves fed milk replacers instead of milk (from healthy cows) do not benefit from this training and defence provided by the colostrum microbiome, so the inclusion of probiotics may help alleviate this deficit.

Like the colostrum microbiota, at birth, the calf’s GIT is dominated by Proteobacteria, Firmicutes, Actinobacteria, and Bacteroidetes, which is similar to the in-utero microbiota, but it can change rapidly. Within 24 h, the calf’s GIT can be colonized by pathogenic bacteria such as *Escherichia, Shigella*, *Clostridium* spp., and *Enterococcus* spp. [417]. This is particularly likely if colostrum administration is delayed [418]. From the results obtained by Malmuthuge et al. [399], calves are born with low bacterial density but with a high proportion of beneficial bacteria, such as *Lactobacillus* and *Bifidobacterium*, compared to potentially pathogenic bacteria, such as *E. coli*. However, colostrum can modulate this microbiome, and in this study, providing colostrum reduced the intestinal *Lactobacillus* content. However, when colostrum was heat-treated (60 °C/60 min), the prevalence of *Bifidobacterium* was higher at 6 h post-natal compared to untreated colostrum or no colostrum groups; however, at 12 h post-natal, there were no differences in *Bifidobacterium* prevalence between the groups of calves that received colostrum. In addition, calves that did not receive colostrum had higher levels of *E. coli* at 6 h and 12 h after birth than calves fed colostrum, especially when compared to calves fed heat-treated colostrum [399]. This confirms that environmental bacteria can colonize the calf’s gut very quickly and highlights the importance of providing good quality (chemically and microbiologically) colostrum soon after birth. During the first few days, the microbiota of calves continues to change and is related to the calf’s health status [414]. At 14 days of age, there were differences in the fecal microbiome between healthy and diarrheal calves, with a high prevalence of *Faecalibacterium* and *Butyricicoccus* species in healthy calves [414]. Colostrum and milk microbiome, in combination with the environment, modulate the GIT microbiome of calves; however, this area of study is relatively new and warrants further investigation. 

## 6. Conclusions

Different considerations of colostrum are found across studies, which makes it difficult to make adequate comparisons between studies, especially in areas where information is limited. Colostrum, transition milk, and mature milk have different physical and chemical proprieties, so it is important to properly define what colostrum is in research activities. Each species has its own unique differences in colostrum composition. The value of bovine colostrum for animal (other than calves) and human consumption has increased due to its nutritional and nutraceutical value but also due to its availability on farms when compared to colostrum from other species. This demand could affect the availability of good quality colostrum for calves, making it important to better understand colostrogenesis and how to improve colostrum quality and quantity, hopefully. From a nutritional and thermoregulatory point of view, high-density colostrum should be fed to newborn calves as soon as possible, as their energy (mainly in the form of fat) and protein requirements are very high. Minerals and vitamins are micronutrients present in colostrum that are essential for normal biological functions. However, there have not been many recent studies, and many of the reported concentrations would benefit from more recent studies using updated techniques. Colostrum also has many bioactive constituents that play multiple roles in the newborn calf. They may act as mediators in the passive transfer of immunity, defend against pathogens, contribute to cellular differentiation and growth, and possibly act as maternal–offspring signaling molecules. However, only a few have been studied. The most studied to date are immunoglobulins (especially IgG), growth factors (mainly IGF-I and IGF-II), and fatty acids (mainly n-3 and n-6 fatty acids). More recently, other bioactive components such as lactoferrin, oligosaccharides, and microRNAs have received more attention. However, there is still a lack of research on some components, such as many hormones whose concentrations in colostrum are not known, as well as the role of nucleic acids, enzymes, cytokines, hormones, and amino acids in newborns. Most, if not all, of these components appear to be important for the newborn calf, as some of them have significantly higher concentrations in colostrum or in colostrum and transition milk compared to whole milk. Cells in colostrum are important modulators of the calf’s immune system but have not been properly studied; they appear to lose viability during the freezing process, which is nefarious as, for practical reasons, many calves receive thawed colostrum from a colostrum bank. Finally, research into the colostrum microbiota has increased recently and its role in colonizing the neonatal gut is crucial for a better understanding of the digestive and absorptive processes of colostrum components, as well as its role in neonatal enteric disease. As morbidity and mortality rates in dairy calves remain high during the first days of life, research into the components and properties of colostrum could help improve calf health and welfare. Future areas of study could focus on colostrum proteomics and metabolomics, oligosaccharides, fatty acids, miRNAs, immune cells, and the microbiome. This opens research opportunities, as bovine colostrum can be used not only for the newborn calf but also as a nutraceutical and therapeutic agent for animals and humans.

## Figures and Tables

**Table 1:** Variability of the main components of bovine colostrum in relation to breed, region, and method of analysis.

Component	Mean	S.D.	Min	Max	CV	*n*	Breed	Region	Method of Analysis	Reference
Total Solids (%)	23.9	3.41			14.3	10	H	Kansas, USA	AOAC	[54]
	24.2					36	H	Cairo, Egypt	AOAC, 1999	[55]
	27.2	5.8	12.9	47.2	21.3	365	H	Isfahan, Iran	Milkoscan	[52]
	27.6	5.84	18.3	43.3	21.2	55	H	Pennsylvania, USA	AOAC, 1975	[56]
	25.8	4.68			18.1	1074	H	Northern Greece	Brix	[53]
	24.7	0.51			2.1	72	H	Central Denmark	Milkoscan	[57]
	26.3		13.5	37		288	HF	Ontario, Canada and Minnesota, USA	Brix	[58]
	25.3	4.9	11.0	42.0	19.4	709	HF	Évora, Portugal	Brix	[59]
	24.8	0.26	18	31	1.0	73	F	Pisa, Italy	Brix	[60]
	22.6	4.7	1.7	33.1	20.8	496	H + J	USA	Milkoscan	[61]
	22.5	6.74			30.0	16	J	Kansas, USA	AOAC	[54]
	27.7	8.76			31.6	99	J	Northern India	Gravimetric	[62]
	23.6	5.56	12.8	36.6	23.6	86	J	USA	Dried overnight	[18]
	23.4	0.74			3.2	32	J	Central Denmark	Milkoscan	[57]
	21.2	4.43	10.5	28.6	20.9	58	J	Iowa, Canada	Brix	[63]
	24.3		10.4	52.6		569		Alberta, Canada	Brix	[45]
Protein (%)	14.0	2.59			18.5	8	H	Kansas, USA	Subtration	[54]
	16.6					6	H	Tuscia, Italy (TN)	Milkoscan 104	[64]
	13.9					6	H	Tuscia, Italy (HT)	Milkoscan 104	[64]
	13.5					36	H	Cairo, Egypt	AOAC, 1999	[55]
	14.9	3.32	7.10	22.6	22.3	55	H	US-PA	Kjeldahl	[56]
	18.5	4.9	4.90	29.6	26.5	365	H	Isfahan, Iran	Milkoscan	[52]
	17.8	3.97			22.3	1074	H	Northern Greece	Milkoscan	[53]
	14.7	3.51	4.55	25.22	23.9	559	H	Northen Italy	Kjeldahl	[65]
	15.4	0.42			2.7	72	H	Central Denmark	Milkoscan	[57]
	14.0	3.67			26.2	1226	H + F *	Northen Ireland	Milkoscan	[66]
	12.6	2.9	3.34	17.12	23.0	76	HF	Swiszterland	Milkoscan	[67]
	18.2	3.94	11.28	24.6	21.6	21	HF	Germany	Milkoscan	[67]
	16.1	1.64			10.2	8	F	Reading, UK	DairyLab II (NIR)	[68]
	12.7	3.3	2.60	20.5	26.0	542	H + J	USA	Milkoscan	[61]
	13.1	4.08			31.1	99	J	Northern India	Kjeldahl	[62]
	23.6	5.07	9.16	31.63	21.5	88	J	USA	Kjeldahl	[18]
	14.2	5.26			37.0	11	J	Kansas, USA	Subtration	[54]
	14.6	0.62			4.2	32	J	Central Denmark	Milkoscan	[57]
Fat (%)	6.7	2.65			39.6	29	H	Kansas, USA	Babcock	[54]
	6.0					6	H	Tuscia, Italy (TN)	Milkoscan 104	[64]
	5.9					6	H	Tuscia, Italy (HT)	Milkoscan 104	[64]
	8.0					36	H	Cairo, Egypt	Gerber	[55]
	6.7	4.16	2.0	26.5	62.1	54	H	US-PA	Babcock	[56]
	4.6	3.4	0.3	20.9	73.9	365	H	Isfahan, Iran	Milkoscan	[52]
	6.4	3.3			51.6	1074	H	Northern Greece	Milkoscan	[53]
	5.2	0.34			6.5	72	H	Central Denmark	Milkoscan	[57]
	4.6	3.04	0.12	14.95	66.1	557	H	Northen Italy	VDLUFA, 2013	[65]
	6.4	3.32			51.9	1226	H + F *	Northen Ireland	Milkoscan	[66]
	4.4	1.75	2.16	8.78	39.8	21	HF	Germany	Milkoscan	[67]
	5.5	2.8	1.32	14.21	50.9	76	HF	Swiszterland	Milkoscan	[67]
	3.55	1.82			51.3	8	F	Reading, UK	DairyLab II (NIR)	[68]
	5.6	3.2	1.0	21.1	57.1	531	H + J	USA	Milkoscan	[61]
	3.3		0.1	8.7			J	USA	Infrared	[18]
	3.4	0.51			15.0	32	J	Central Denmark	Milkoscan	[57]
	4.2	1.81			43.1	32	J	Kansas, USA	Babcock	[54]
	8.0	7.96			99.5	99	J	Northern India	Gerber	[62]
Lactose (%)	2.7	0.91			33.7	8	H	Kansas, USA	AOAC 1945	[54]
	3.2					6	H	Tuscia, Italy (TN)	Milkoscan 104	[64]
	2.6					6	H	Tuscia, Italy (HT)	Milkoscan 104	[64]
	2.5	0.65	1.2	5.2	26.0	55	H	US-PA	Colorimetric	[56]
	2.0	0.9	0.3	5.2	45.0	365	H	Isfahan, Iran	Milkoscan	[52]
	3.68	0.04			1.1		H	Central Denmark	Milkoscan	[57]
	2.15	0.73			34.0	1074	H	Northern Greece	Milkoscan	[53]
	2.36	0,51	0.74	4.06	21.6	577	H	Northen Italy	HPLC	[65]
	2.7	0.55			20.4	1226	H + F *	Northen Ireland	Milkoscan	[66]
	3.2	0.53	1.94	4.6	16.6	76	HF	Swiszterland	Milkoscan	[67]
	2.9	0.59	1.82	3.81	20.3	21	HF	Germany	Milkoscan	[67]
	2.7	0.46			17.0	8	F	Reading, UK	DairyLab II (NIR)	[68]
	2.9	0.5	1.2	4.6	17.2	538	H + J	USA	Milkoscan	[61]
	3.73	0.06			1.6	32	J	Central Denmark	Milkoscan	[57]
	2.4	0.77			32.1	11	J	Kansas, USA	AOAC 1945	[54]
	3.0	0.20			6.7	99	J	Northern India	Lane-Eynon	[62]
Minerals (%)	1.11	0.16			14.4	10	H	Kansas, USA	Evaporation	[54]
	0.05	0.01	0.02	0.07	20.0	55	H	US-PA	AOAC, 1975	[56]
	1.9	0.17			8.9	8	F	Reading, UK	DairyLab II (NIR)	[68]
	1.02	0.40			39.2	99	J	Northern India	Incineration	[62]
	1.22	0.14			11.5	16	J	Kansas, USA	Evaporation	[54]

We only considered values from first milking after birth. When not provided, SD (standard deviation) was calculated from SE (standard error) with the following formula: SD = SE × Sqrt(*n*). CV—Coefficient of variation was calculated based on the mean and SD reported in each reference. Total solids, dry matter, and brix were considered the same component. Missing values were not reported. TS, Total Solids; H, Holstein breed; F, Friesian breed; HF, Holstein–Friesian breed; J, Jersey breed. * Mostly Holstein and Friesian. H, F, and HF differentiation: The Holstein–Friesian breed started as Friesian in Northern Europe in the second half of the XVIII century; after the XIX century, Friesian cows were exported to Canada and the United States of America, where they were renamed Holstein–Friesian and Holstein, respectively. Different genetic traits were selected in the continents of Europe and America; in Europe, animals were selected for mixed traits (milk and meat production), while in America, animals were selected purely for milk capacity [69]. All three names, “Friesian”, “Holstein”, and “Holstein–Friesian” can be found in the literature. In the analysis presented in this table, Friesian cows were only used in European countries, while the others were used in different continents. The identification has been done according to the information provided by the authors in the methodology section of the paper.

**Table 3:** Mean concentrations and physiological roles of vitamins present in the bovine colostrum.

Vitamin	Mean	Physiological Role
Fat-soluble vitamins		
Vitamin A (µg/100 mL)	233–369	Immune function, cell-growth, and vision.
Vitamin E (µg/100 g)	191–530	Antioxidant function.
Vitamin D (IU/100 g fat)	120–181	Ca and P absorption, bone health, and immune function.
Vitamin K (µg/100 mL)	>2	Blood clotting and bone health.
Water-soluble vitamins		
Thiamine (B1) (µg/100 mL)	58–90	Energy metabolism and nervous system.
Riboflavin (B2) (µg/100 mL)	455–610	Energy production and cell growth.
Niacin (B3) (µg/100 mL)	34–96	Redox reactions (synthesis of NAD), energy metabolism.
Pantothenic acid (B5) (µg/100 mL)	224	Acetyl-transfer reactions (synthesis of coenzyme A), energy metabolism.
Pyridoxal (B6) (µg/100 mL)	15.0	Brain development, immune function, and production of hemoglobin.
Pyridoxamine (B6) (µg/100 mL)	21.0	
Pyridoxine (B6) (µg/100 mL)	4.0	
Biotin (B7) (µg/100 mL)	1.0–2.7	Carboxylation reactions, glucose, amino acids, and fatty acids metabolism.
Folate (B9) (µg/100 mL)	0.75–0.8	Single-carbon-transfer reactions (nucleic acids synthesis), DNA, and methionine metabolism.
Cobalamin (B12) (µg/100 mL)	0.2–60	Red blood cell production, neurological function, and DNA synthesis.
Vitamin C (µg/100 mL)	1620–3200	Antioxidant, immune function, skin, and blood vessel integrity.

Values represent the range of mean concentrations and were obtained from refs [7,22,56,143,144,145,146,147,148].

**Table 4:** Predominant trends of fatty acids in colostrum compared to transitional milk or milk.

Fatty Acid	Predominant Trend
C4:0 Butyric Acid	↓
C6:0 Caproic Acid	↓
C8:0 Octanoic Acid	?
C12:0 Lauric Acid	?
C14:0 Myristic acid	↑
C14:1 ω-5 Myristoleic acid	?
C15:0 pentadecanoic acid	?
C16:0 Palmitic acid	↑
C16:1 ω-7 Palmitoleic	?
C17:0 Heptadecanoic acid	?
C18:0 Stearic acid	?
C18:1 ω-9 Oleic acid	?
C18:2 ω-6 Linoleic acid (LA)	↑
C18:3 ω-3 α-Linolenic acid (ALA)	?
C21:0 Behenic acid	?
C20:3 ω-6 Dihomo-γ-linolenic acid	?
C23:0 Tricosanoic acid	?
SFA	?
Branched-chain FA	?
MUFA	?
Trans-MUFA	↓
Conjugated linoleic acid (CLA)	↓
PUFA	↑
ω-3	↑
ω-6	↑

↓ Indicates that the fatty acid or fatty acid group is present at a lower concentration in colostrum than in transitional milk or milk; ? indicates that the results are inconclusive; only one author has presented results or there is an inconsistency between references; ↑ indicates that the fatty acid or fatty acid group is present at a higher concentration in colostrum than in transitional milk or milk. Based on refs [27,227,228].

**Table 5:** Fatty acids present in higher concentration (g/100 g total fatty acid) in colostrum compared to milk (at 5th day of lactation).

Fatty Acid	Colostrum	Milk (5 d)
C14:0 Myristic acid	12.8–13.7	8.3–11.2
C16:0 Palmitic acid	32.5–40.4	27.2–29.7
C18:2 ω-6 Linoleic acid (LA)	1.95–2.79	1.53–2.23
PUFA	3.88–4.28	2.97–3.62
ω-3	0.56–1.18	0.33–0.70
ω-6	2.57–3.72	2.64–3.00

Values represent the range of mean concentrations and were obtained from refs [27,227,228].

**Table 6:** Most abundant sialylated oligosaccharides in bovine colostrum and milk with corresponding concentrations (mg/L).

Oligosaccharide	Raw Colostrum	Mature Milk
3′-Sialyllactose (3′SL)	341–867	42–114
6′-Sialyllactose (6′SL)	103–243	17–89
6′-Sialyllactosamine (6′SLN)	117–239	11–170
Disialyllactose (DSL)	84–520	4–38

Values represent the range of mean concentrations and were obtained from refs [24,256,257,258,259,260].

**Table 8:** Mean variation in the differential leukocyte count (%) in bovine colostrum and milk.

Differential Leukocyte Count	Colostrum ^a^	Milk ^b,^*
Lymphocytes (total)	2–27	18–58
Lymphocytes T	16	47
Lymphocytes B	11	20
Macrophages	31–69	10–29
Neutrophils	30–65	28–49

Values represent the range of mean concentrations. ^a^ Values were obtained from refs [347,373,377,379]; ^b^ Values were obtained from refs [382,383,384]; * Includes results from dairy cows in various stages of lactation.

**Table 9:** Core microbiome of colostrum (genus level), according to the frequency that a certain genus has been considered part of the colostrum core microbiome in the literature.

Genus	Phyla	Frequency
*Acinetobacter*	Proteobacteria	+++++
*Pseudomonas*	Proteobacteria	++++
*Staphylococcus*	Firmicutes	++++
*Bacteroides*	Bacteroidetes	+++
*Corynebacterium*	Actinobacteria	+++
*Streptococcus*	Firmicutes	+++
*Bacillus*	Firmicutes	++
*Chryseobacterium*	Bacteroidetes	++
*Flavobacterium*	Bacteroidetes	++
*Lactococcus*	Firmicutes	++

In terms of inclusion criteria, the genera have to be designated as part of the colostrum core or top genera in at least two studies. The symbol “+” indicates the number of studies. Values were obtained from refs [14,410,411,413,414].

## Data Availability

The original contributions presented in the study are included in the article/supplementary material, further inquiries can be directed to the corresponding author.

## Supplementary Materials

The following supporting information can be downloaded at: https://www.mdpi.com/article/10.3390/ani14071130/s1, Table S1: Qualitative comparison of lipid content between colostrum and either transitional or mature milk. Table S2: Most abundant sialylated oligosaccharides in bovine colostrum and corresponding concentrations (mg/L). Mean values from each reference are shown, as well as a calculated mean, standard deviation (SD), and coefficient of variation (CV). Figure S1: Box plot of the range of macronutrient concentrations in colostrum. The graphic was constructed with the means reported in Table 1. Figure S2: Dot-plot of the range of major protein concentrations in colostrum. Dots from the same study have the same colour. Figure S3: Dot-plot of the range in the minor protein concentrations in colostrum. Dots from the same study have the same colour. Figure S4: Dot-plot of the range in the major oligosaccharide’s concentrations in colostrum.
