# Supplementary material for: A Comprehensive Review of Bovine Colostrum Components and Selected Aspects Regarding Their Impact on Neonatal Calf Physiology
Source: Animals (Basel). 2024 Apr 8;14(7):1130. doi: 10.3390/ani14071130 (PMC11010951; doi:10.3390/ani14071130)
Supplement: Supplementary file 1 [file animals-14-01130-s001.zip › animals-2924796-supplementary.pdf]

Supplementary file for "A Comprehensive Review of Bovine Colostrum Components and Selected Aspects Regarding Their Impact on Neonatal Calf Physiology".

Supplementary Table S1. Qualitative comparison of lipid content between colostrum and either transitional or mature milk

**Fatty acids**	**Lower**	**Similar**	**Higher**
C4:0 Butyric Acid	W, O		
C6:0 Caproic Acid	W, O		
C8:0 Octanoic Acid	O		
C12:0 Lauric Acid			O
C14:0 Myristic acid			W, O
C14:1 ω-5 Myristoleic acid			O
C15:0 Pentadecanoic acid			O
C16:0 Palmitic acid			W, O
C16:1 ω-7 Palmitoleic			O
C17:0 Heptadecanoic acid	O	W	
C18:0 Stearic acid	O		
C18:1 ω-9 Oleic acid	O		
C18:2 ω-6 Linoleic acid (LA)			W, O
C18:3 ω-3 α-Linolenic acid (ALA)		W	O
C21:0 Behenic acid		O	
C20:3 ω-6 Dihomo-γ-linolenic acid			O
C23:0 Tricosanoic acid			O
Saturated fatty acids		W, C	O
Unsaturated fatty acids	O		
Branched-chain FA	W		
MUFA	O	W	
Trans-MUFA	W C		
PUFA			W, O,
Conjugated linoleic acid (CLA)	C, O		
ω-3			C, O, W
ω-6		C	W, O
Cholesterol			C, P
Phospholipids (total)			C
Phosphatidylethanolamine	C,		
Phosphatidylcholine		C,	
Phosphatidylserine		C	
Phosphatidylinositol		C	
Sphingomyelin			C
Triglycerides (48-52 carbons)			C
Triglycerides (24-36 carbons)	C		

"Lower" and "Higher" indicate a significant difference in the concentration of a fatty acid or fatty acid group between colostrum and transition milk or milk. "Similar" indicates that there were no significant differences. Letters (P, C, O and W) refer to each reference where significant differences were observed between the fatty acid or fatty acid group concentration in colostrum compared to transition milk or milk.
O, O'Callaghan et al. [226]; W, Wilms et al. [27]; C, Contarini et al. [227]; P, Precht [228].

Supplementary Table S2. Most abundant sialylated oligosaccharides in bovine colostrum and corresponding concentrations (mg/L). Mean values from each reference are shown, as well as a calculated mean, standard deviation (SD), and coefficient of variation (CV).

OS	Ref. 1*	Ref. 2	Ref. 3	Ref. 4†	Ref. 5	Ref. 6	Mean	SD	CV (%)
3′SL	834; 2388	594	853	681; 867	787	341	918	619	67
6′SL	144; 222	103	141	243;136	113	112	152	52	34
6′SLN	228; 534	145	117	239;220	-	204	241	137	57
DSL	84; 336	225	-	201;238	520	131	248	144	58

Some values may not actually refer to colostrum but to transition milk, as in some experiments "colostrum" was not collected until the 2nd day of lactation. Some values were obtained using graphical analysis software (https://plotdigitizer.com/app) when no numerical values were given.
OS, Oligosaccharide; 3′SL, 3′-sialyllactose; 6′SL, 6′-sialyllactose; 6′SLN, 6′-sialyllactosamine; DSL, disialyllactose; * fresh (left) and heat-treated (right) colostrum are shown; † Friesian (left) and Jersey (right) cows.
Refs. 1 to 6: [258], [257], [255], [259], [24], and [256], respectively.

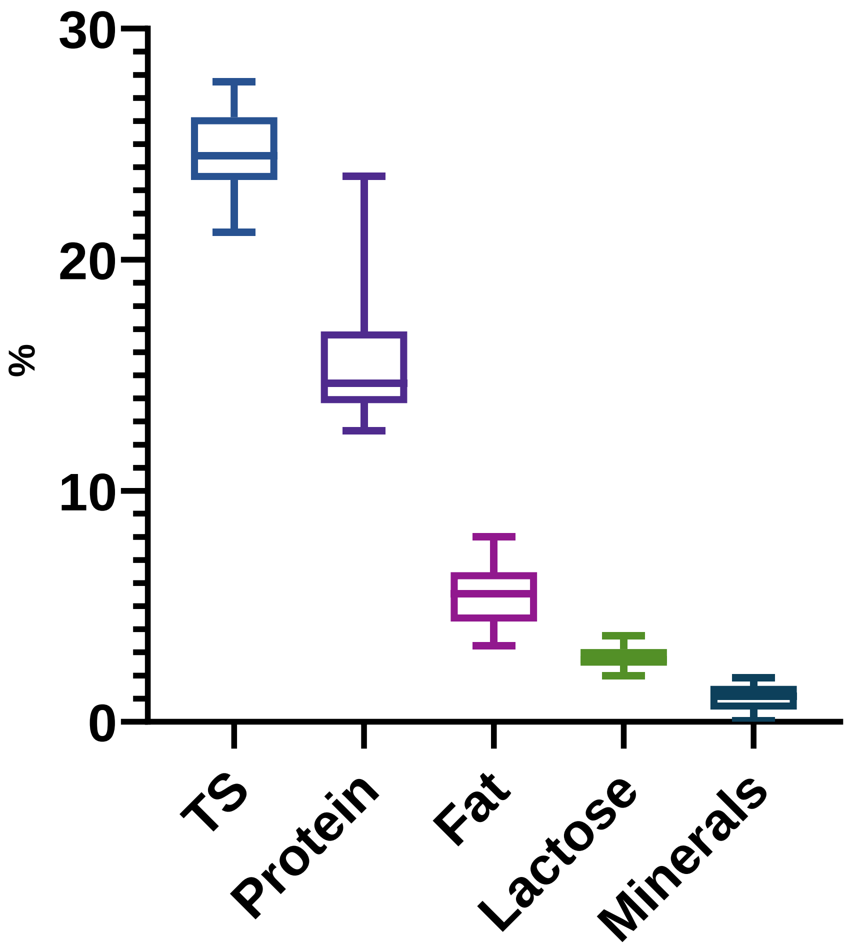

Supplementary Figure S1. Box plot of the range of macronutrient concentrations in colostrum. The graphic was constructed with the means reported in Table 1. Graphic created with GraphPad Prism 9.

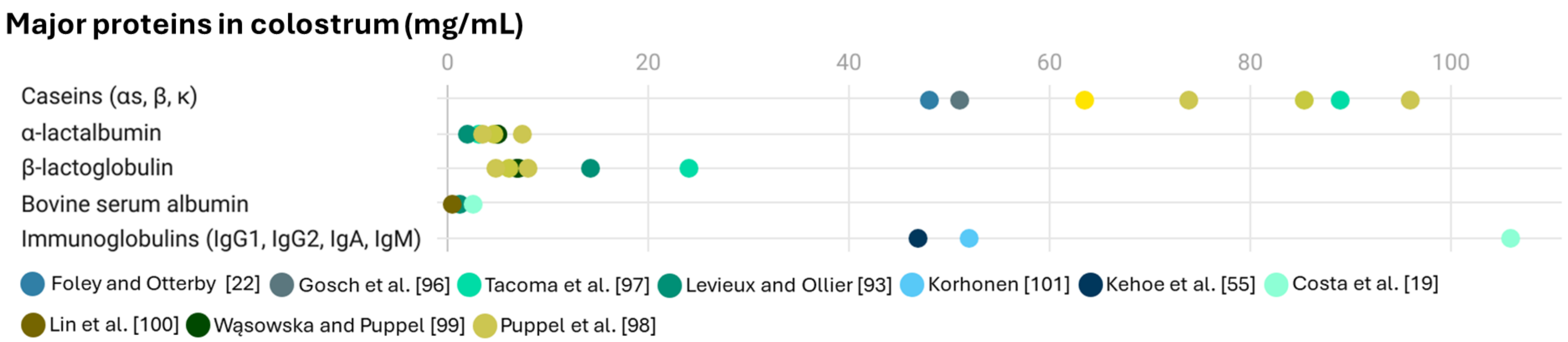

Supplementary Figure S2. Dot-plot of the range of major protein concentrations in colostrum. Dots from the same study have the same colour. Graphic created with DataWrapper (https://www.datawrapper.de) and adapted with Microsoft Power Point and Paint.

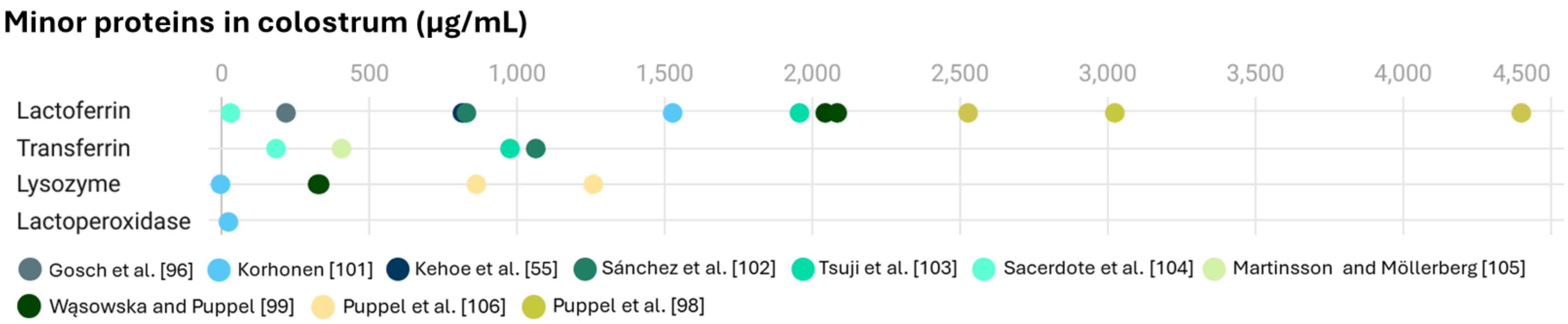

Supplementary Figure S3. Dot-plot of the range of minor protein concentrations in colostrum. Dots from the same study have the same colour. Graphic created with DataWrapper (https://www.datawrapper.de) and adapted with Microsoft Power Point and Paint.

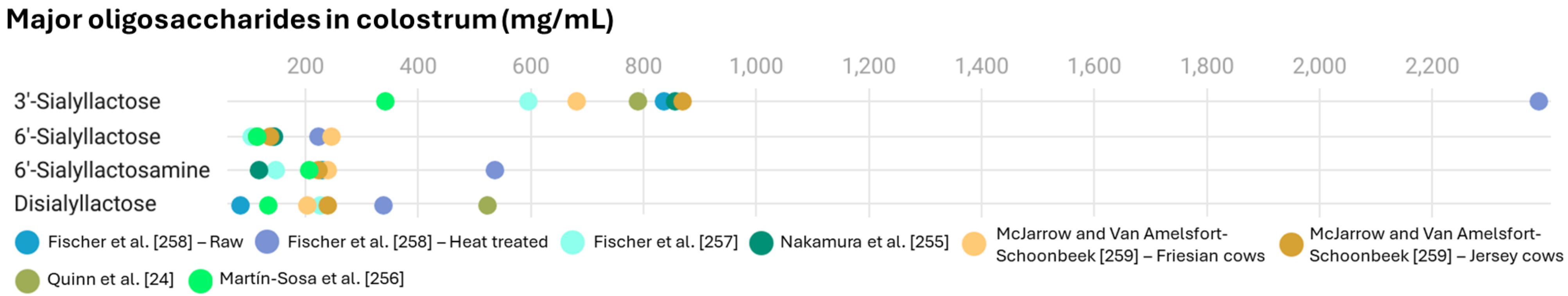

Supplementary Figure S4. Dot-plot of the range in the major oligosaccharide's concentrations in colostrum. Dots from the same study have the same colour. When a study presented different values showing the effect of a certain factor a variation of the colour is represented and the treatment is referred by the reference. Graphic created with DataWrapper (https://www.datawrapper.de) and adapted with Microsoft Power Point and Paint.
